# Lysosomes drive the piecemeal removal of mitochondrial inner membrane

**DOI:** 10.1038/s41586-024-07835-w

**Published:** 2024-08-21

**Authors:** Akriti Prashar, Claudio Bussi, Antony Fearns, Mariana I. Capurro, Xiaodong Gao, Hiromi Sesaki, Maximiliano G. Gutierrez, Nicola L. Jones

**Affiliations:** 1 Cell Biology Program, The Hospital for Sick Children; Toronto, Canada; 2 Host-Pathogen Interactions in Tuberculosis Laboratory, The Francis Crick Institute; London, UK; 3 Department of Cell Biology, Johns Hopkins University School of Medicine; Baltimore, USA; 4 Division of Gastroenterology, Hepatology and Nutrition, The Hospital for Sick Children; Toronto, Canada; 5 Departments of Paediatrics and Physiology, University of Toronto; Toronto, Canada

## Abstract

Mitochondrial membranes define distinct structural and functional compartments. Cristae of the inner mitochondrial membrane (IMM) function as independent bioenergetic units that undergo rapid and transient remodeling, but the significance of this compartmentalized organization is unknown^1^ . Using super-resolution microscopy, here we show that cytosolic IMM vesicles, devoid of outer mitochondrial membrane or mitochondrial matrix, are formed during resting state. These vesicles derived from the IMM (VDIMs) formed by IMM herniation through pores formed by the voltage-dependent anion channel (VDAC1) in the outer mitochondrial membrane. Live-cell imaging showed that lysosomes in proximity to the mitochondria engulfed the herniating IMM, and aided by the ESCRT machinery, led to the formation of VDIMs in a microautophagy-like process, sparing the rest of the organelle. VDIM formation was enhanced in mitochondria undergoing oxidative stress, suggesting their potential role in maintaining mitochondrial function. Furthermore, the formation of VDIMs required calcium release by the ROS activated, lysosomal calcium channel, transient receptor potential mucolipin1 (TRPML1), showing an inter-organelle communication pathway for maintaining mitochondrial homeostasis. Thus, IMM compartmentalization could allow for the selective removal of damaged IMM sections via VDIMs, which should protect mitochondria from localized injury. Our findings reveal a new pathway of intramitochondrial quality control.

Mitochondrial homeostasis is maintained by the hierarchical and coordinated activity of several quality control (QC) pathways. These include the removal of the entire organelle by processes like mitophagy and mitocytosis, as well as mitochondrial remodeling by membrane sorting pathways for removal of selective cargo (mitochondria derived vesicles-MDVs, mitochondria derived compartments-MDCs, structures positive for mitochondrial outer membrane-SPOTs and mitochondria-lysosome-related organelle-MLRO)^2–6^.

The classical view of mitochondrial organization suggests that a uniform membrane potential (ΔΨ_m_) exists across the entire organelle and membrane depolarization leads to the clearance of damaged mitochondria by mitophagy^1^. However, recent findings have demonstrated that unlike this equipotential model, each individual crista is an independent, highly dynamic, anatomic and functional unit that maintains its own ΔΨ_m_, independently of adjacent cristae^7,8^. The inner mitochondrial membrane (IMM) is sub-compartmentalized into the inner boundary membrane (IBM), running parallel to the outer mitochondrial membrane (OMM), and the cristae, formed by IMM invaginations^9^. Cristae junctions (CJs) separate the cristae from the IBM and delineate specialized compartments where the complexes needed for ATP generation by oxidative phosphorylation are localized^9^. This compartmentalized IMM organization is speculated to prevent the spread of damage from one crista to the rest of the organelle^7^. However, localized damage could still cause mitochondrial dysfunction if damaged and healthy membranes mix during mitochondrial fusion, or if reactive oxygen species (ROS) produced by impaired cristae damage mitochondrial DNA (mtDNA). Therefore, we hypothesized that mechanisms must exist to selectively remove the impaired sections of the IMM, without causing damage to the entire organelle. By studying the remodeling of mitochondrial membranes using super-resolution microscopy, we discovered the presence of distinct, cytosolic IMM-derived vesicles that are encapsulated by lysosomes. Mechanistically, these vesicles form when IMM herniates through VDAC pores in the OMM and is engulfed by lysosomes in a microautophagy like process, requiring the ESCRT (endosomal sorting complex required for transport) machinery. Our findings reveal that IMM compartmentalization can facilitate the selective removal of damaged IMM sections, thereby serving as an intramitochondrial QC mechanism.

## Cells form VDIMs at steady state

**ED Fig. 1:**
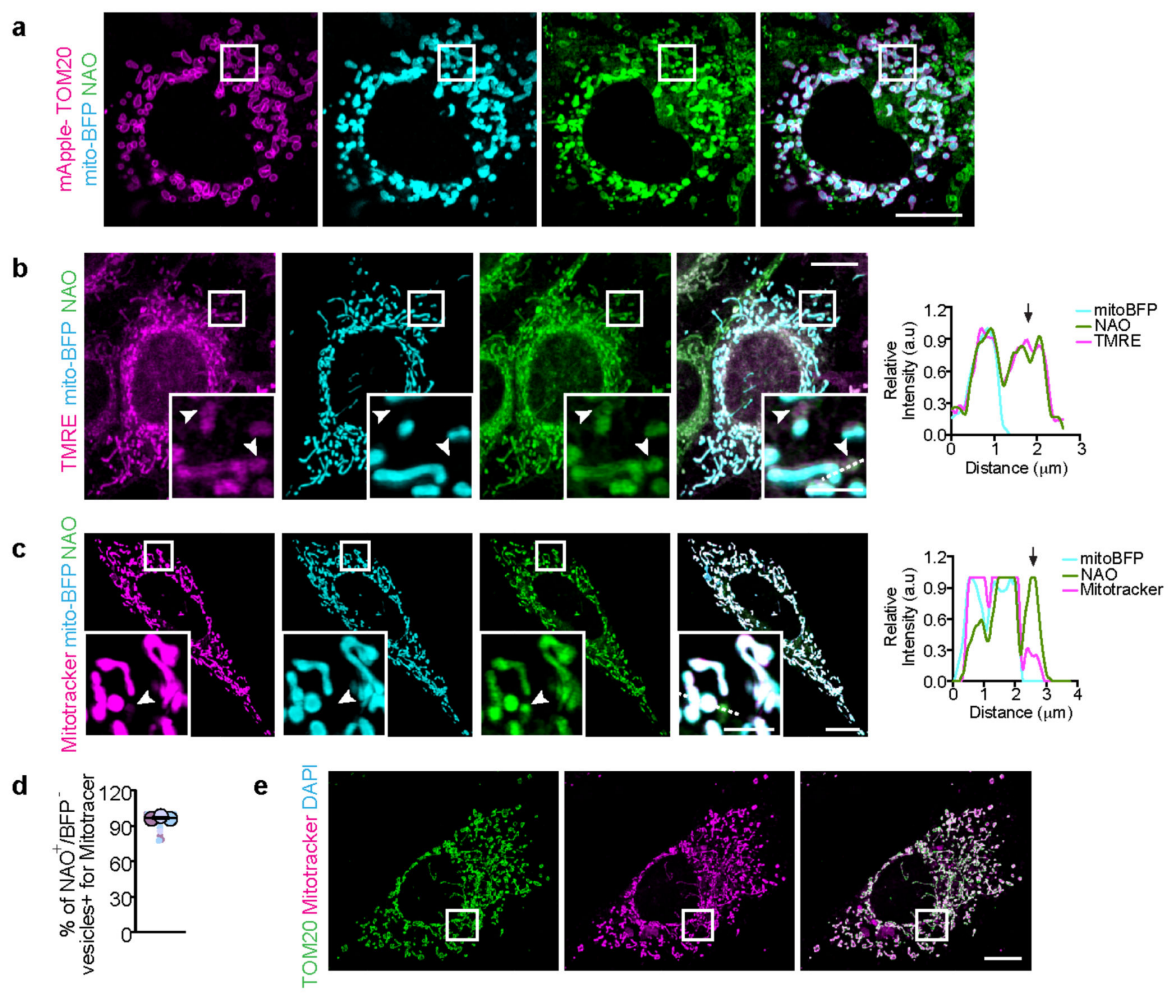
Cytosolic IMM-derived vesicles lack matrix and OMM **a**, TOM20 and NAO localization. Inner mitochondrial membranes were labeled with NAO (green) in cells expressing mito-BFP (cyan) and mApple-TOM20 (magenta). Higher magnifications of indicated regions are shown in Fig.1a. **b**, Localization of TMRE (magenta), NAO (green) in cells expressing mito-BFP (cyan). Higher magnifications of indicated regions are shown. Arrowheads indicate mito-BFP^-^/NAO^+^/TMRE^+^ vesicles. **Right**: Pixel intensity plots for dashed line. Arrow indicates the vesicle. **c**, NAO (green), mitotrackerCMXRos (mitotracker) (magenta) localization in cell expressing mito-BFP (cyan). Higher magnifications of indicated regions are shown. Arrowheads indicate mito-BFP^-^/NAO^+^/mitotracker^+^ vesicle. **Right**: Pixel intensity plots for dashed line. Arrow indicates the vesicle. **d**, Percentage of vesicles positive for IMM markers NAO and mitotracker, but negative for mito-BFP from experiments as in (**c**) (n=365 vesicles, 30 cells, 3 experiments). Data shown are mean±SEM shown as large circles and individual data points from corresponding experiments are shown in the same color (**e**) Representative images from at least three independent experiments showing mitotracker (magenta) and TOM20 (green) localization. Higher magnifications of indicated regions are shown in Fig.1c. Scale bars: main panels10μm, magnified panels 3μm.

**ED Fig. 2:**
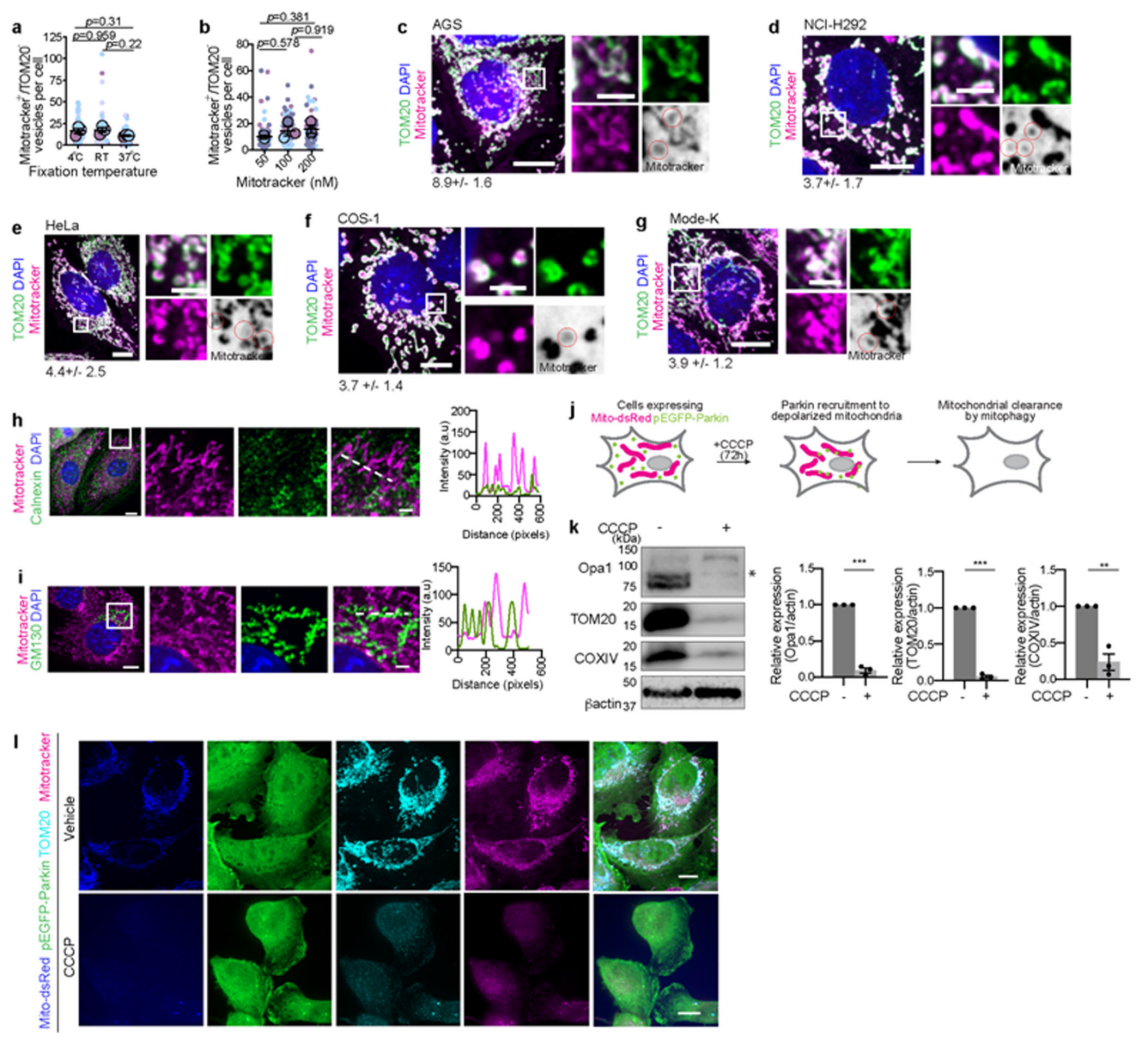
Validating specificity of IMM in VDIMs **a**, Effect of fixation conditions on mitotracker^+^/TOM20^-^ vesicles. Cells labeled with mitotracker (100nM, 15min, 37^o^C) were either fixed for 15 min at room temperature (RT), 4^o^C overnight or at 37^o^C in pre-warmed 4% paraformaldehyde for 10min (n=60 cells, 3 experiments). **b**, Effect of mitotracker concentration on mitotracker^+^/TOM20^-^ vesicles. Cells were stained with indicated concentrations of mitotracker (n= 62 cells for 50nM, 64 cells for 100 nM, 53 cells for 200nM, 3 experiments). **c-g**, Mitotracker^+^/TOM20^-^ vesicles are cell-type independent. Spinning disc confocal images of mitotracker^+^/TOM20^-^ vesicles in (**c**) AGS (n=4 experiments), (**d**) NCI-H292 (n=4 experiments), (**e**) HeLa (n=2 experiments), (**f**) COS-1 (n=2 experiments), and (**g**) Mode-K (n=3 experiments) cells. For all images, numbers at the bottom indicate the number of vesicles (mean±SEM), and higher magnifications from indicated regions from main panels are shown to the right. Inverse color micrographs for mitotracker channel are shown. Red circles indicate the mitotracker^+^/TOM20^-^ vesicles. **h**,**i**, Mitotracker does not label membranes non-specifically. Spinning disc confocal images showing localization of mitotracker (magenta) and ER specific Calnexin (green) (**h**), or Golgi specific GM130 (green) (**i**). **Right**: Pixel intensity plot for dashed lines. **j**, Schematic illustrating the protocol for depleting mitochondria from HeLa cells stably expressing Mito-dsRed and pEGFP-Parkin. **k**, Western blot showing the depletion of mitochondrial proteins in cells from (**j**). **Right**: Protein expression relative to actin. **l**, Spinning disc confocal images showing lack of mitochondrial labeling by mitotracker (magenta) in HeLa cells expressing mito-dsRed (blue) and pEGFP-Parkin (green), labeled with anti-TOM20 antibodies (cyan) in an experiment as in (**j**). Data shown are mean±SEM from three independent experiments. Statistical significance was calculated using One-way ANOVA followed by Tukey’s multiple-comparison test in (**a-b**), and two-tailed Student’s unpaired t-test in (**k**). *P* values calculated are shown. Gel source data for (**k**) are provided in Supplementary Fig.1. Scale bars: main panels 10μm, magnified panels 3μm.

**Fig. 1:**
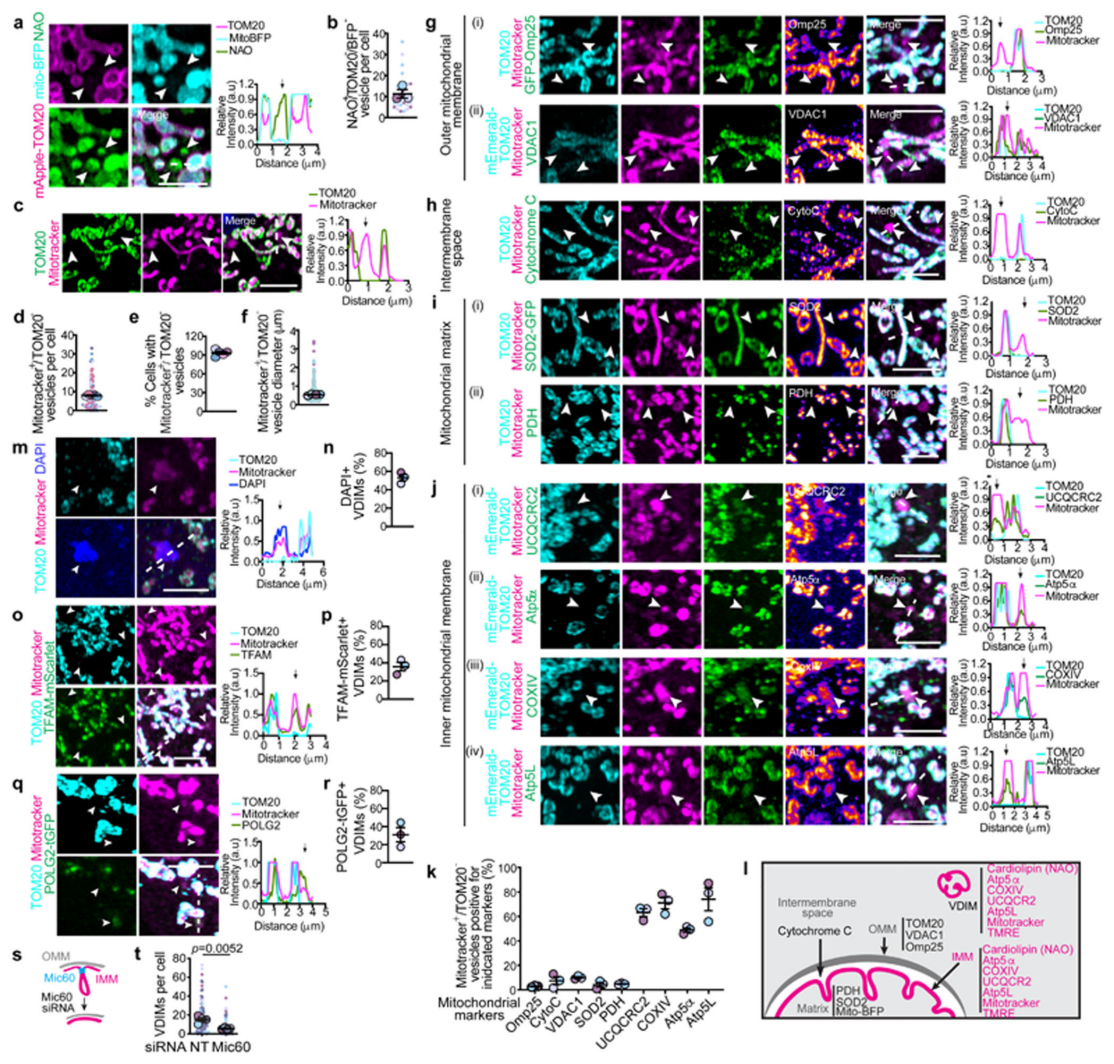
Selective sorting of mitochondrial inner membrane proteins into VDIMs **a**, Cytosolic TOM20^-^ /NAO^+^/ mito-BFP^-^ vesicles (arrowheads) in MEFs expressing mito-BFP (cyan, matrix) and mApple-TOM20 (magenta, OMM), stained with NAO (green, IMM). **Right:** Pixel intensity plot for dashed line. Arrow indicates the vesicle. **b**, Number of TOM20^-^/ NAO^+^/mito-BFP^-^ vesicles in experiments as in (**a**) (n=336 vesicles, 30 cells, 3 experiments). **c**, Representative images of mitotracker^+^/TOM20^-^ vesicles (arrowheads). **Right:** Pixel intensity plot for dashed line. Arrow indicates the vesicle. **d**,**e**, Quantification of (**d**) number of mitotracker^+^/TOM20^-^ vesicles (n=824 vesicles, 104 cells, 5 experiments), and (**e**) percentage of cells containing mitotracker^+^/TOM20^-^ vesicles (n=104 cells, 5 experiments) in experiments as in (**c**). **f**, Size of mitotracker^+^/TOM20^-^ vesicles in experiments as in (**c**) (n=548 vesicles, 42 cells, 4 experiments). **g**, Lack of localization of OMM localized proteins (i) GFP-Omp25 (green), or (ii) VDAC1 (green) with mitotracker^+^/TOM20^-^ vesicles. **h**, Lack of localization of intermembrane space localized cytochrome C (green) with mitotracker^+^/TOM20^-^ vesicles. **i**, Lack of localization of mitochondrial matrix localized proteins (i) SOD2-GFP (green) or (ii) PDH (green) with mitotracker^+^/TOM20^-^ vesicles. **j**, Presence of IMM localized proteins, (i) UCQCRC (green), (ii) Atp5α (green), (iii) COXIV (green) or (iv) Atp5L (green) in mitotracker^+^/TOM20^-^ vesicles in cells expressing m-Emerald-Tom20 (cyan). **k**, Percentage of mitotracker^+^/TOM20^-^ vesicles positive for indicated mitochondrial markers from (**g-j**) (n=349 vesicles, 30 cells, 3 experiments for Omp25; n=282 vesicles, 31 cells, 3 experiments for VDAC1; n=312 vesicles, 33 cells, 3 experiments for cyto C; n=288 vesicles, 30 cells, 3 experiments for SOD2; n=294 vesicles, 30 cells, 3 experiments for PDH; n=286 vesicles, 29 cells, 3 experiments for UCQCRC2; n=216 vesicles, 30 cells, 3 experiments for COXIV; n=210 vesicles, 31 cells, 3 experiments for Atp5α; n=267 vesicles, 30 cells, 3 experiments for Atp5L). **l**, Schematic showing the mitochondrial markers analyzed for their localization with mitotracker^+^/TOM20^-^ vesicles. **m-r**, Presence of mtDNA in VDIMs. **m**, VDIMs in cells stained with DAPI. **Right:** Pixel intensity plot for dashed line. Arrow indicates the vesicle. **n**, Percentage of VDIMs positive for DAPI in experiments as in (**m**) (n= 442 vesicles, 70 cells, 3 experiments). **o**, VDIMs (arrowheads) in cells expressing TFAM-mScarlet (green). **Right:** Pixel intensity plot for dashed line. Arrow indicates the vesicle. **p**, Percentage of VDIMs positive for TFAM in experiments as in (**o**) (n=484 vesicles, 30 cells, 3 experiments). **q**, VDIMs (arrowheads) in cells expressing POLG2-tGFP (green). **Right:** Pixel intensity plot for dashed line. Arrow indicates the vesicle. **r**, Percentage of VDIMs positive for POLG2 in experiments as in (**q**) (n=347 vesicles, 30 cells, 3 experiments). **s**, Schematic illustrating the effect of silencing Mic60 on IMM organization. **t**, Number of VDIMs in cells treated with non-targeting (NT) or Mic60 siRNA (n=123 cells for NT, 121 cells for Mic60 siRNA, 4 experiments). (**e, k, n, p, r**) Data shown are mean±SEM. (**b, d, f, t)** Mean±SEM are shown as large circles and individual data points from corresponding experiments are shown in the same colors. Statistical analysis was performed using two-tailed Student’s unpaired t-test. *P* values are indicated. **(b, e, k, n-r)** Quantifications from Airyscan images. **(g-j)** Pixel intensity plots for dashed lines are shown to the right. Arrow indicates the vesicles. Rainbow pseudo-colored images for the indicated mitochondrial markers are shown. Representative images from three independent experiments are shown. All scale bars: 3μm

To study the different mitochondrial compartments, we labeled mitochondria in immortalized mouse embryonic fibroblasts (MEFs) at steady state with the mitochondria selective dye nonyl acridine orange (NAO) that partitions to the IMM^7^, along with matrix targeted mito-BFP and OMM localized mApple-TOM20. We observed distinct cytosolic NAO^+^ (IMM) vesicles that lacked matrix or OMM (Fig. 1a-b, Extended data Fig. 1a). These NAO^+^ vesicles were also detected using additional IMM localized probes tetramethylrhodamine, ethyl ester (TMRE) and MitoTracker CMXRos^7^ (herein referred to as mitotracker) (Extended Data Fig. 1b-d). Since, mitotracker is well retained after fixation, and the formation of these vesicles was not affected by fixation conditions (Extended Data Fig. 2a), or mitotracker concentration (Extended Data Fig. 2b), we utilized mitotracker and TOM20, which was absent from these vesicles (Fig. 1c, Extended Data Fig. 1e) to further investigate their formation.

**ED Fig. 3:**
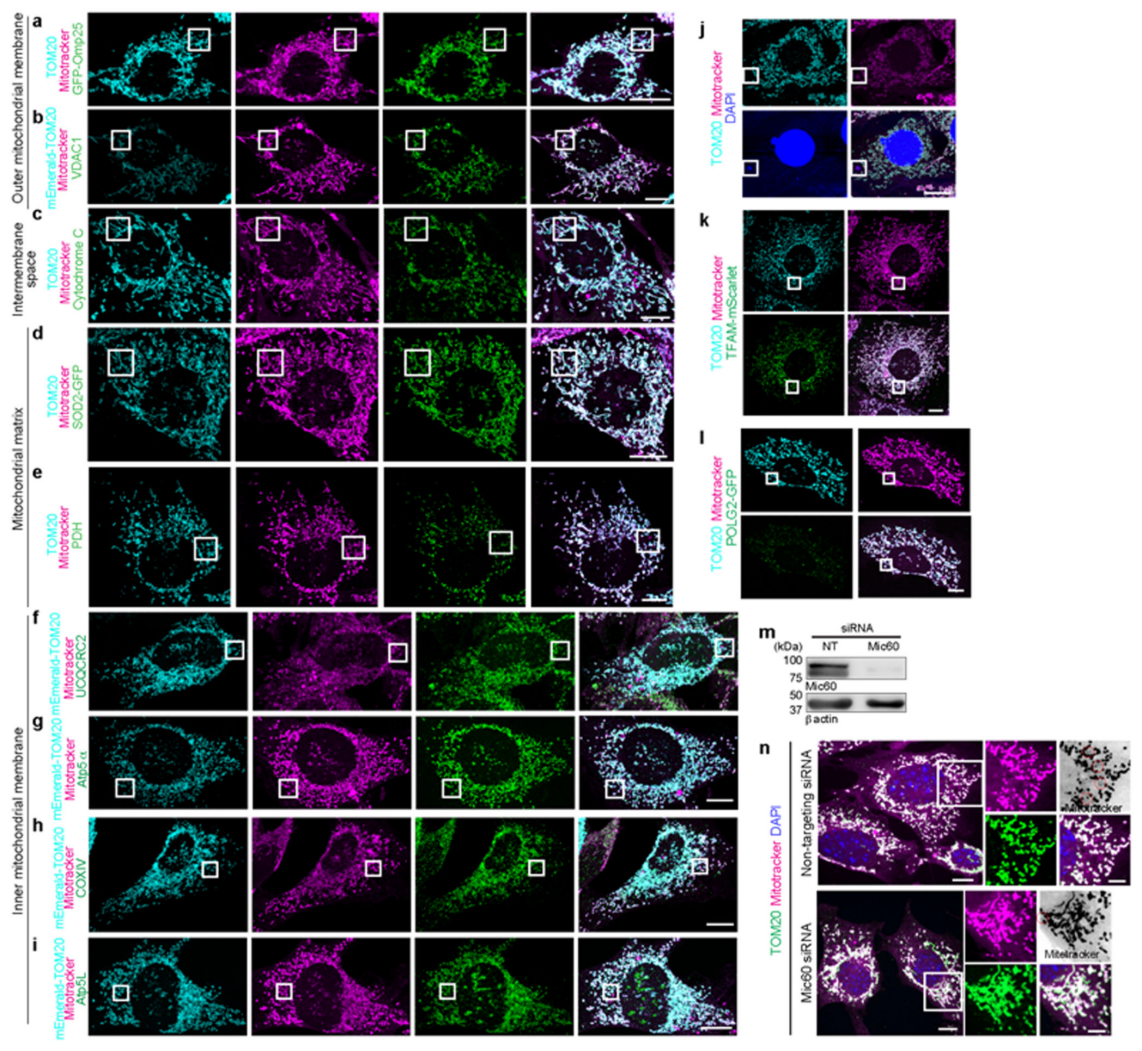
VDIMs are derived from the IMM **a-i**, Localization of mitotracker along with markers associated with different mitochondrial compartments. **a**,**b**, Representative images showing localization of mitotracker (magenta) and OMM localized (**a**) Omp25 (green) and TOM20 (cyan), or (**b**) VDAC1 (green) and mEmerald-TOM20 (cyan). Higher magnifications of the indicated regions are shown in Fig.1g. **c**, Representative images showing localization of mitotracker (magenta) and mitochondrial intermembrane space-localized cytochrome C (green). OMM was labeled with anti-TOM20 antibodies (cyan). Higher magnifications of the indicated regions are shown in Fig.1h. **d**,**e**, Representative images showing localization of mitotracker (magenta), TOM20 (cyan) and mitochondrial matrix-localized (**d**) SOD2 (green) and (**e**) PDH (green). Higher magnifications of the indicated regions are shown in Fig.1i. **f-i**, Representative images showing localization of mitotracker (magenta) in cells expressing mEmerald-TOM20(cyan) and mitochondrial IMM-localized (**f**) UCQCRC2, (**g**) Atp5α, (**h**) COXIV and (**i**) Atp5L (green). Higher magnifications of the indicated regions are shown in Fig1j. (**j**) Representative images showing MEFs labeled with mitotracker (magenta), anti-TOM20 antibodies (cyan) and high concentration of DAPI (5μg/ml). Higher magnifications of indicated regions are shown in Fig.1m. **k**, Localization of TOM20 (cyan) and mitotracker (magenta) in cells expressing TFAM-mScarlet (green). Higher magnifications of indicated regions are shown in Fig.1o. **l**, Localization of TOM20 (cyan) and mitotracker (magenta) in cells expressing POLG2-tGFP (green). Higher magnifications of indicated regions are shown in Fig.1q. **m**, Representative western blot showing the efficiency of Mic60 knockdown. Cells were treated with indicated siRNA. **n**, VDIM formation in cells treated with indicated siRNA. Mitochondria were labeled with mitotracker (magenta) and anti-TOM20 antibodies (green). Representative confocal images from three independent experiments (**a**-**l**) and four independent experiments (**n**) are shown. Gel source data for (**m**) are provided in Supplementary Fig.1. Scale bars: main panels 10μm, magnified panels 3μm. Red circles in the inverted mitotracker micrographs indicate the VDIMs.

At steady state >90% of cells contained the mitotracker^+^/TOM20^-^ vesicles, with an average of 7.9 vesicles per cell and an average diameter of ∼0.5μm (Fig. 1d-f). Importantly, these vesicles were present at resting state in all cell types tested (Extended data Fig. 2c-g). We next excluded the possibility that these vesicles were membranes non-specifically labelled by mitotracker. Mitotracker did not localize with ER or Golgi specific markers, Calnexin or GM130, respectively (Extended Data Fig. 2h-i). Furthermore, in cells where mitochondria were severely depleted^10^, mitotracker labeling was not detected (Extended Data Fig. 2j-l). Consistent with IMM labeling, the mitotracker^+^/TOM20^-^ vesicles contained makers from the IMM but lacked proteins localized at the OMM, mitochondrial matrix or the intermembrane space (Fig. 1g-k, Extended Data Fig. 3a-i), confirming their IMM origin. We refer to these IMM-derived vesicles that lack markers from all other mitochondrial compartments as VDIM (Vesicles Derived from the Inner Mitochondrial membrane) (Fig. 1l). A proportion of VDIMs contained mtDNA as shown by labelling with the DNA specific fluorescent dye DAPI (4′,6-diamidino-2-phenylindole), and mtDNA specific TFAM (transcription factor A) and POLG2 (DNA polymerase subunit gamma-2) (Fig. 1m-r, Extended Data Fig. 3j-l).

We reasoned that if the compartmentalized organization of the IMM facilitates the selective removal of IMM by VDIMs, then altering this organization should impede VDIM formation. The complex architecture of the IMM is regulated by the MICOS (mitochondrial contact site and cristae organizing system) complex, consisting of seven subunits, including Mic60, which is necessary to stabilize the complex and maintain IMM organization and mitochondrial function^8,11^. In line with this reasoning, VDIM formation was significantly impaired in cells depleted of Mic60 (Fig.1s-t, Extended data Fig.3m-n), indicating that IMM compartmentalization contributes to their formation, and further supporting the findings that VDIMs arise from the IMM.

**ED Fig. 4:**
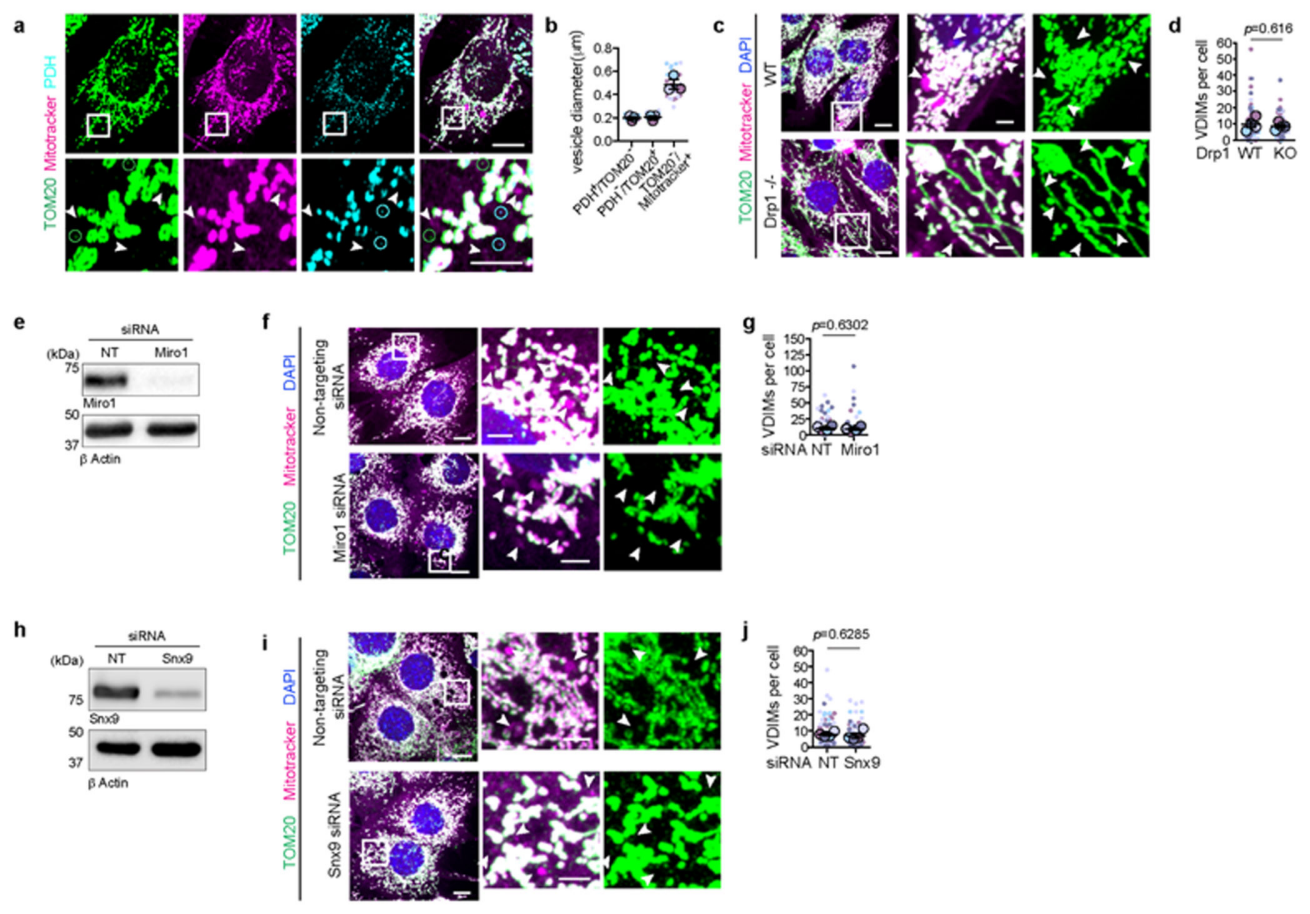
VDIMs are distinct from MDVs and MDCs **a**, VDIMs are larger than MDVs. Size difference between the Tom20 (green) or PDH (cyan) positive mitochondria derived vesicles (MDVs) (indicated by open circles), and mitotracker^+^/TOM20^-^ vesicles (arrowheads). **Bottom**: Higher magnifications of indicated regions. **b**, Size difference between the Tom20 (green) or PDH (cyan) positive mitochondria derived vesicles (MDVs) (indicated by open circles), and mitotracker^+^/TOM20^-^ vesicles (arrowheads) in experiments as in (**a**) (n= 562 vesicles, 30 cells, 3 experiments for PDH^+^/TOM20^-^ vesicles; n=605 vesicles, 30 cells, 3 experiments for PDH^-^/TOM20^+^ vesicles; n=436 vesicles, 30 cells, 3 experiments for mitotracker^+^/TOM20^-^ vesicles). **c**, Representative spinning disc confocal images showing VDIM formation in WT and Drp1^**-/-**^ MEFs. Arrowheads indicate VDIMs. **d**, Number of VDIMs in WT and Drp1^-/-^ (KO) MEFs in experiments as in (**c**) (n= 80 cells, 4 experiments). **e**, Representative western blot (n=4 experiments) showing loss of Miro1 expression in cells treated with non-targeting (NT) siRNA or siRNA against Miro1. **f**, Representative spinning disc confocal images showing VDIM formation in cells treated with NT or Miro1 siRNA an experiment as in (**e**). Arrowheads indicate VDIMs. **g**, Number VDIMs in cells treated with non-targeting (NT) or Miro1 siRNA in experiments as in (**f**) (n= 90 cells, 4 experiments). **h**, Representative western blot (n=4 experiments) showing loss of sorting nexin 9 (Snx9) expression in cells treated with non-targeting (NT) siRNA or siRNA against Snx9. **i**, Representative spinning disc confocal images showing VDIM formation in cells in experiments as in (**h**). **j**, Number of VDIMs in cells treated with non-targeting (NT) or syntaxin 9 (snx9) siRNA in experiments as in (**i**) (n= 90 cells, 4 experiments). Data shown are mean±SEM from three independent experiments. Statistical significance was calculated using two-tailed Student’s unpaired t-test. Gel source data for (**e**,**h**) are provided in Supplementary Fig.1. Scale bars: main panels 10μm, magnified panels 3μm.

The large diameter of VDIMs (Fig. 1f, Extended Data Fig. 4a-b) suggested that they differed from MDVs which are 60-150 nm ^12,13^ Indeed, VDIM formation did not require the activity of Drp1 (dynamin related protein 1) and Miro1 (mitochondrial RHO GTPase 1), GTPases crucial for MDV formation^13^ (Extended Data Fig. 4c-g). Additionally, depletion of SNX9 (sorting nexin 9), which plays a role in the generation and lysosomal delivery of a subset of MDVs^14^, did not alter VDIM formation (Extended Data Fig. 1h-j). Similarly, unlike MDCs, which also require Drp1 for their formation, and are induced in response to amino acid stress ^6,15^, VDIMs were detectable at resting state. Lastly, in contrast to VDIMs, SPOTs are defined as mitotracker ^-^/TOM20^+^ structures^3^. Altogether, these findings demonstrate that VDIMs represent distinct, newly identified structures that originate from the IMM.

## VDIM formation for mitochondrial QC

**ED Fig. 5:**
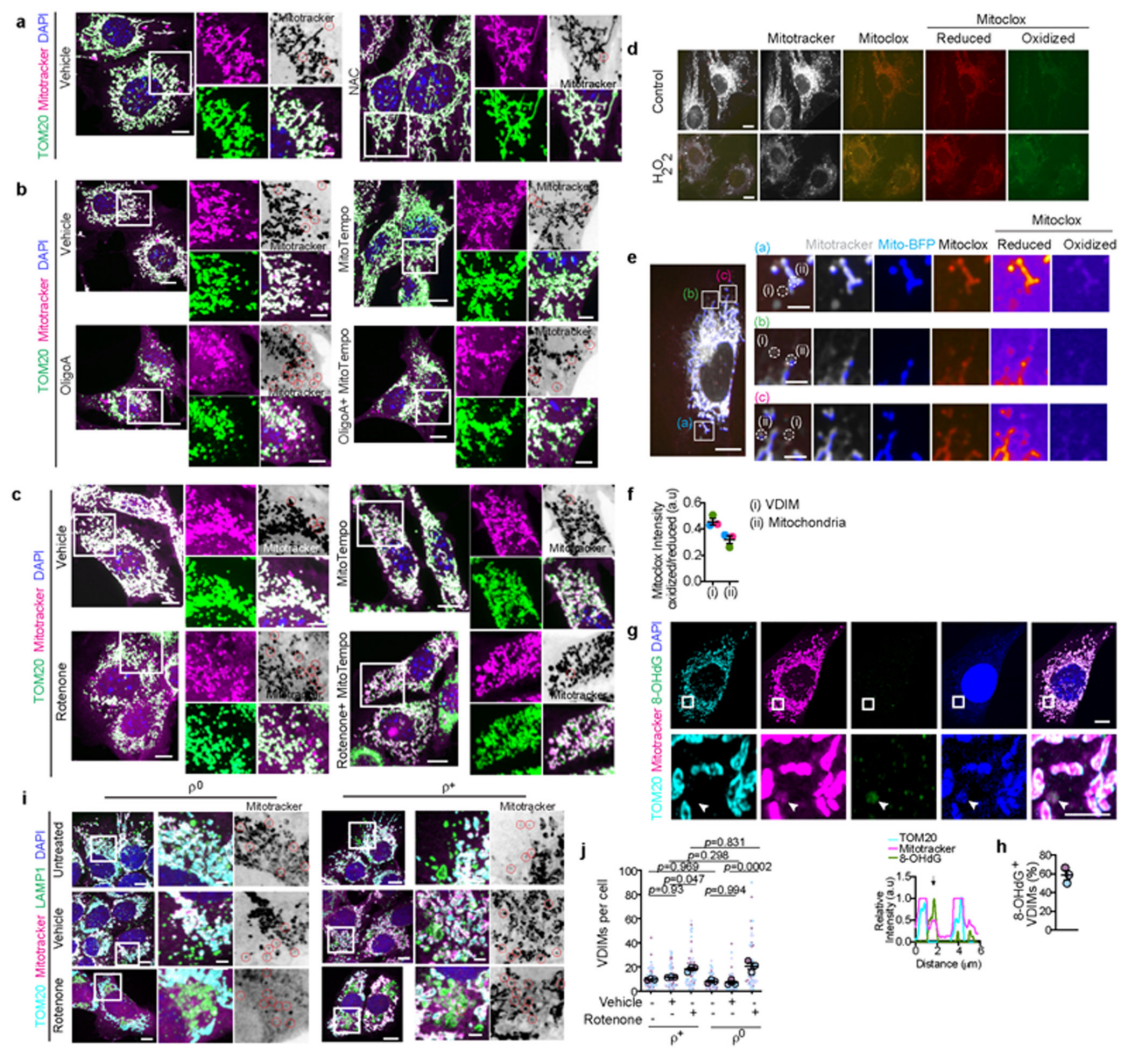
VDIM formation for intramitochondrial QC **a**, VDIM formation is inhibited by quenching ROS. Representative spinning disc confocal micrographs showing the effect of NAC on VDIM formation. Red circles in the inverted color mitotracker micrographs indicate VDIMs. **b**,**c**, Representative confocal micrographs showing the effect of inducing oxidative stress on VDIM formation. Cells were treated with (**b**) oligomycinA, mitoTempo or oligomycinA+mitoTempo, or (**c**) with rotenone, mitoTempo or rotenone+mitoTempo. Mitochondria were labeled with mitotracker (magenta) and anti-TOM20 antibodies (green). Red circles in the inverted color mitotracker micrographs indicate VDIMs. **d**, Representative images showing oxidation of MitoCLox in cells treated with H_2_O_2._ **e**, Representative images showing oxidized MitoCLox in VDIMs (n= 3 experiments). **Right**: Higher magnification of indicated regions. **f**, Ratio of oxidized/total MitoCLox in indicated regions from (**e**). **g**, Localization of 8-OHdG (green) with VDIMs (arrowheads). **Bottom:** Pixel intensity plot for dashed line. Arrow indicates the vesicle. **h**, Percentage of VDIMs positive for 8-OHdG in experiments as in (**g**). (n=272 vesicles, 32 cells, 3 experiments). **i**, Representative spinning disc confocal images showing localization of TOM20 (cyan), mitotracker (magenta) and LAMP1 (green) in 143bρ^0^ and 143bρ^+^ cells. Cells were left untreated or treated with vehicle or Rotenone. Higher magnifications of indicated regions are shown to the right. **j**, Quantification of VDIM formation in 143b cells lacking mitochondrial DNA (ρ0) along with controls (ρ+) (143bρ^0^ n= 70 cells for untreated, 63 cells for vehicle, 60 cells for rotenone and 143bρ^+^ n=66 cells for untreated, 61 cells for vehicle, 60 cells for rotenone, 3 experiments). Data shown are mean±SEM shown as large circles and individual data points from corresponding experiments shown in the same colors. Statistical significance was calculated using One-way ANOVA followed by Tukey’s multiple-comparison test. *P* values calculated are indicated. Scale bars: main panels 10μm, magnified panels 3μm.

**Fig. 2:**
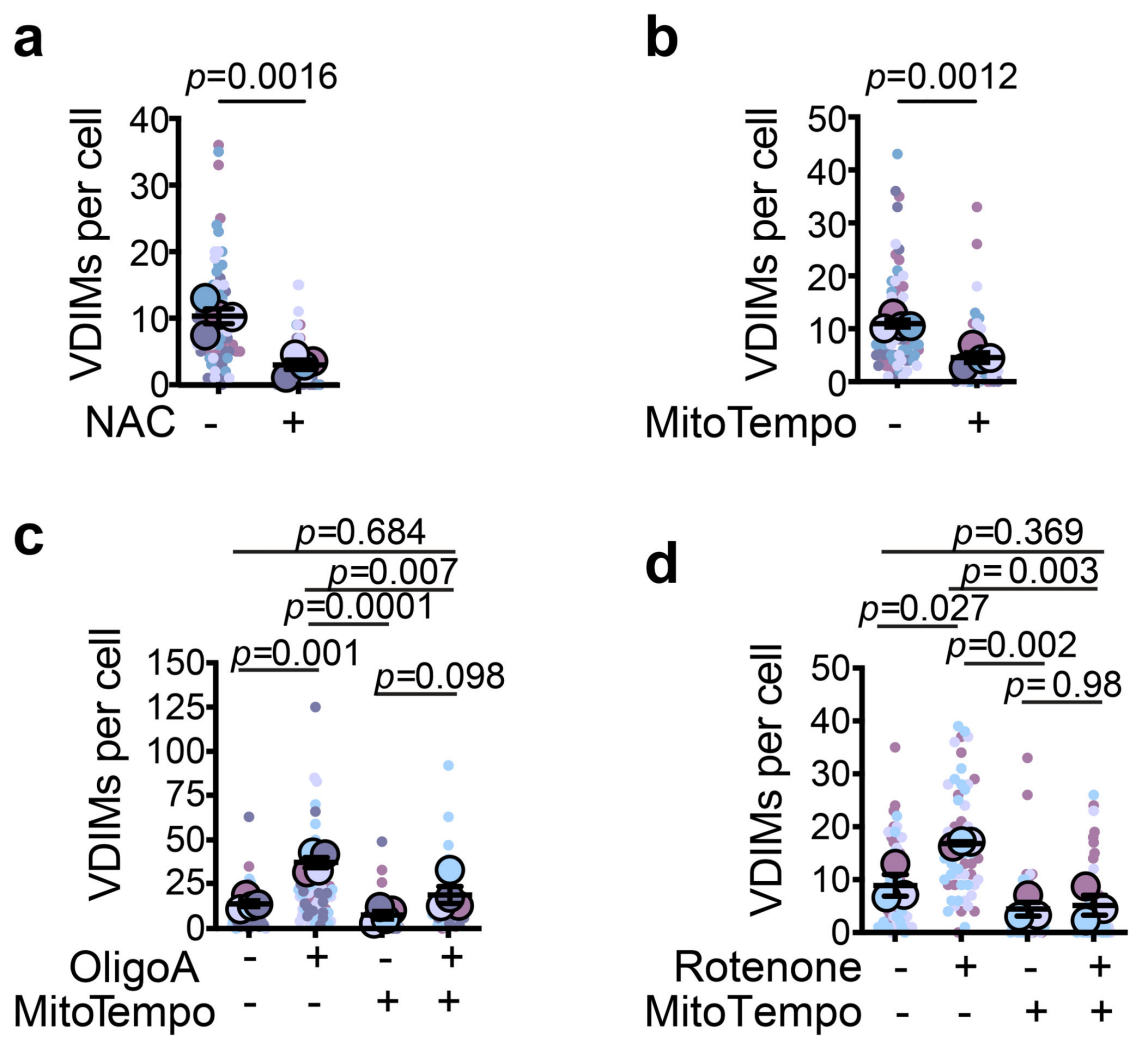
VDIM formation as an intramitochondrial quality control mechanism **a**,**b**, Effect of scavenging ROS on VDIM formation. Cells were treated with (**a)** vehicle (-) and NAC (+) (n=80 cells, 4 experiments), or (**b**) mitoTempo (n=81 cells, 4 experiments). **c**,**d**, Effect of oxidative damage on VDIM formation. **c**, Cells were treated with oligomycinA (oligoA), mitoTempo, or oligoA+mitoTempo (n=82 cells for vehicle, 81 cells for mitoTempo, 79 cells for oligoA, 79 cells for mitoTempo+oligo, 4 experiments), or **d**, rotenone, mitoTempo or rotenone+mitoTempo (n=60 cells for vehicle, 60 cells for mitoTempo, 62 cells for rotenone, 60 cells for rotenone+mitoTempo, 3 experiments). Data shown are mean±SEM shown as large circles and individual data points from corresponding experiments shown in the same colors. Statistical significance was calculated using two-tailed Student’s unpaired t-test in (**a, b**), or One-way ANOVA followed by Tukey’s multiple-comparison test in (**c, d**). *P* values calculated are indicated.

Given that VDIMs lacked both the mitochondrial matrix and OMM proteins, we speculated that they were formed from IMM sections that were selectively removed as a part of a QC mechanism. To determine if VDIMs promote the selective removal of IMM during mitochondrial stress, we first assessed their formation in cells undergoing oxidative stress-mediated mitochondrial damage. Treatment with mitochondrial stressors increased, whereas treatment with antioxidants NAC (N-acetyl-L-cysteine) or mitochondria-targeted MitoTempo markedly reduced VDIM formation (Fig. 2a-d, Extended data Fig. 5a-c). We next assessed whether VDIMs contained damaged IMMs by using the peroxidation sensitive probe MitoCLox, which labels cardiolipin in the IMM^16,17^. Lipid peroxidation, as detected by an increase in green/red intensity (Extended Data Fig. 5d-f) indicated the presence of oxidized cardiolipin in the VDIMs, collectively supporting their role in mitochondrial maintenance.

**ED Fig. 6:**
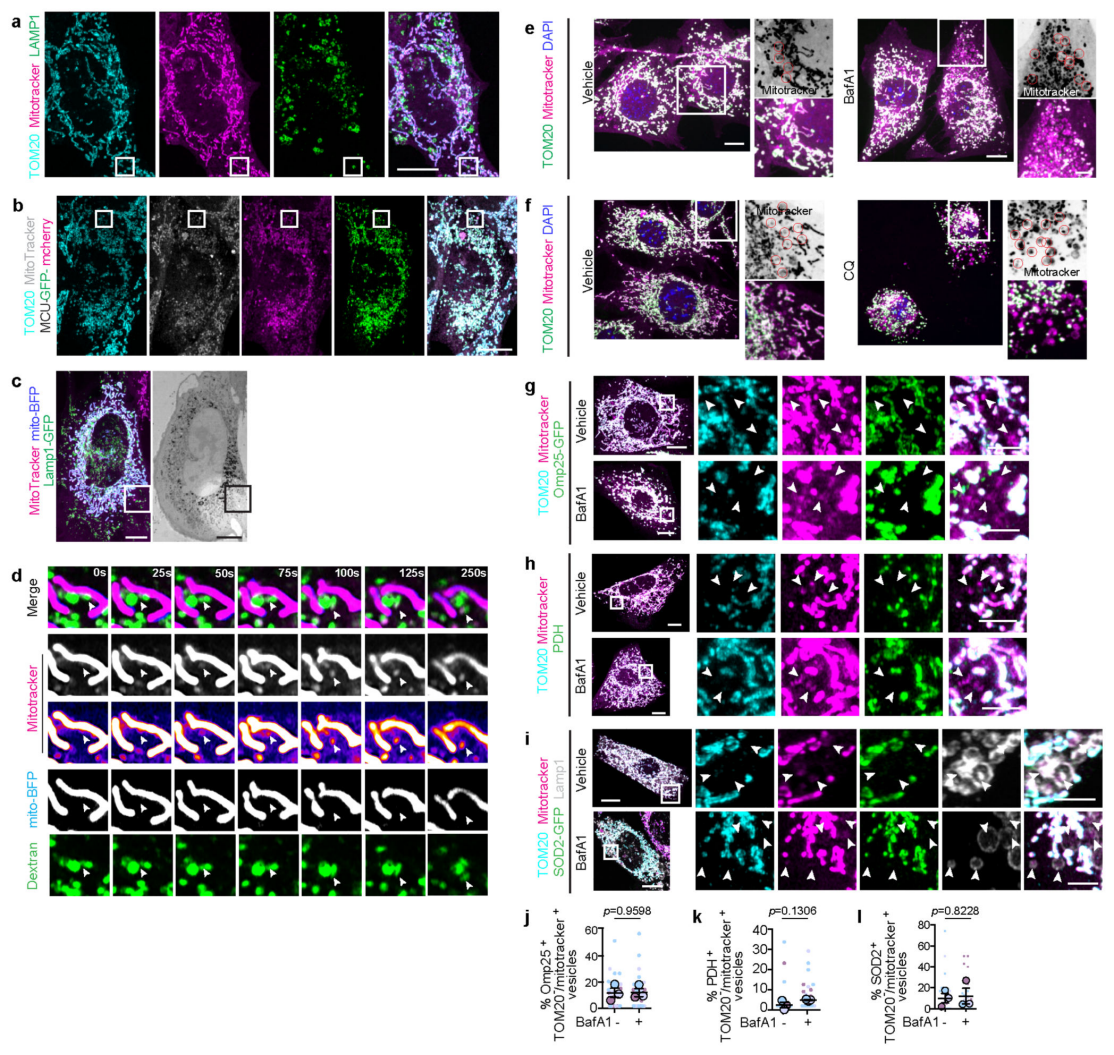
VDIMs are delivered to lysosomes for degradation **a**, Mitotracker (magenta), TOM20 (cyan) and LAMP1 (green) localization. Higher magnifications of indicated regions are shown in Fig.3a. **b**, Localization of mitotracker (grey), TOM20 (cyan) and MCU-GFP-mCherry. Higher magnifications of indicated regions are shown in Fig.3d. **c, Left:** Fluorescence, and **Right:** EM image of cell used for CLEM. Higher magnification of indicated region is shown in Fig. 3f. **d**, Live-cell imaging showing mito-BFP^-^/mitotracker^+^ (magenta) vesicles being delivered to lysosomes labeled with dextran (green). Arrowheads indicate the VDIM pinching from the mitochondria and sorted to the lysosome. Images were acquired every 5sec. **e-l**, VDIMs are not partially degraded mitochondria. **e**, Representative spinning disc confocal micrographs showing the effect of bafilomycin (BafA1) on VDIM formation. **Right**: Higher magnifications of indicated regions. Red circles in inverted micrograph for mitotracker indicate the VDIMs. **f**, Representative spinning disc confocal micrographs showing the effect of chloroquine (CQ) on VDIM formation. **Right**: Higher magnification of indicated regions. Red circles in inverted micrograph for mitotracker indicate the VDIMs. **g**, Localization of mitotracker (magenta), TOM20 (cyan) in cells expressing Omp25-GFP (green), treated with vehicle (-) or BafA1. Arrowheads indicate the VDIMs. (**h**) Localization of mitotracker (magenta), TOM20 (cyan) and PDH (green) in cells treated with vehicle (-) or BafA1. Arrowheads indicate the VDIMs. **i**, Localization of mitotracker (magenta), TOM20 (cyan) in cells expressing SOD2-GFP (green), treated with vehicle (-) or BafA1. Arrowheads indicate the VDIMs. **j**, Percentage of mitotracker^+^/TOM20^-^ VDIMs positive for Omp25 in cells treated with BafA1 compared to vehicle treated cells from experiments as in (**g**) (n= 270 vesicles for vehicle, 503 vesicles for BafA1, 30 cells, 3 experiments). **k**, Percentage of mitotracker^+^/TOM20^-^ VDIMs positive for PDH in cells treated with BafA1 compared to vehicle treated cells from experiments as in (**h**) (n= 270 vesicles for vehicle, 503 vesicles for BafA1, 30 cells, 3 experiments). **l**, Percentage of mitotracker^+^/TOM20^-^ VDIMs positive for SOD2 in cells treated with BafA1 compared to vehicle treated cells from experiments as in (**i**) (n=369 vesicles for vehicle, 500 vesicles for BafA1, 30 cells, 3 experiments). (**j-l**) Mean±SEM are shown as large circles and individual data points from corresponding experiments are shown in the same colors. Statistical analysis was performed using two-tailed Student’s unpaired t-test. *P* values are indicated. Scale bars: main panels 10μm, magnified panels 3μm.

**Fig. 3:**
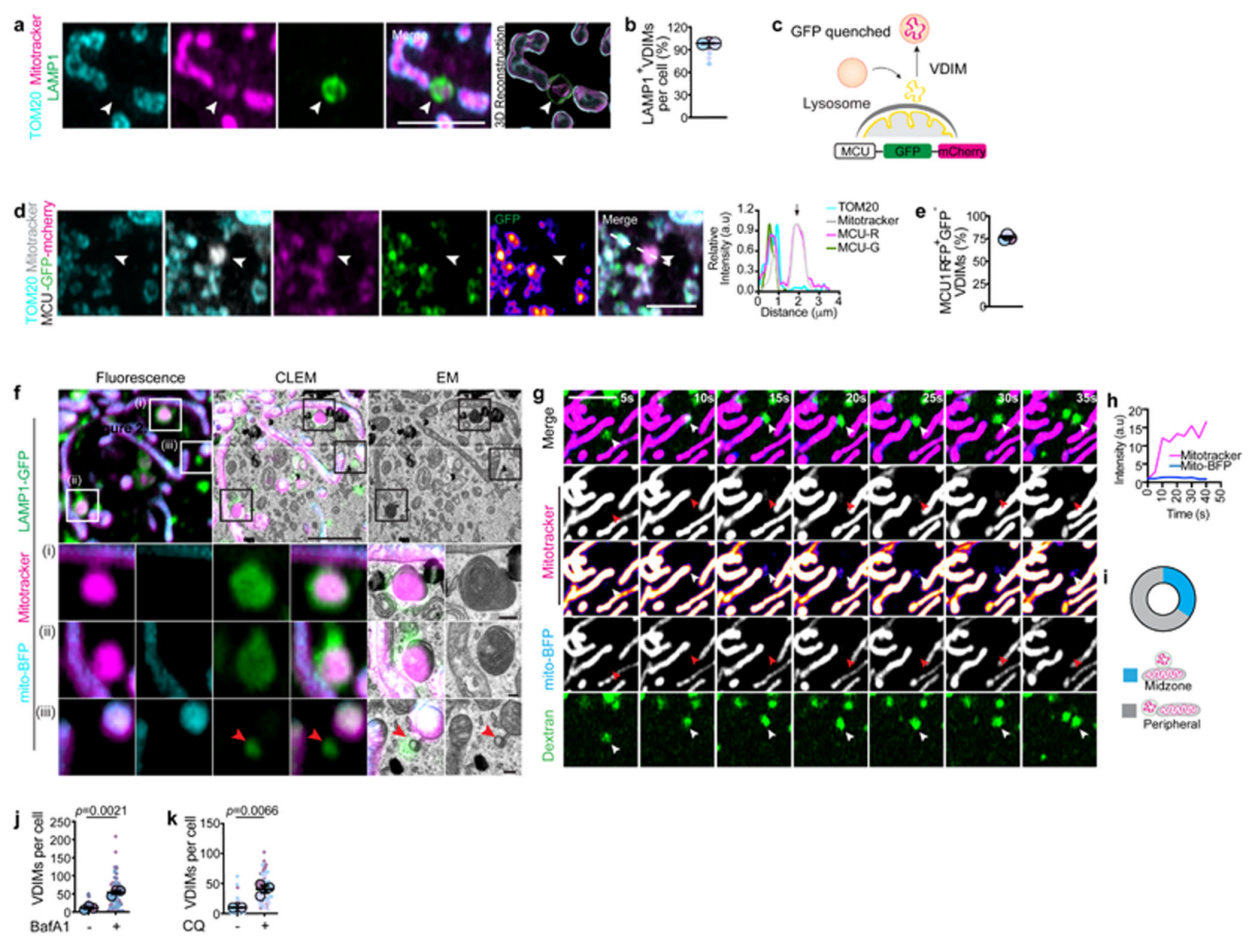
VDIMs are delivered to lysosomes for degradation **a**, Localization of VDIMs in LAMP1^+^ lysosomes. **Right**: 3D reconstruction. Arrowheads indicate the VDIMs. **b**, Number LAMP1positive VDIMs in experiments as in (**a**) (n= 36 cells, 3 experiments). **c**, Schematic illustrating the loss of GFP fluorescence from lysosome-localized VDIMs in cells expressing MCU-GFP-mCherry. **d**, VDIMs in cells expressing MCU-GFP-mCherry. Arrowheads indicate the VDIMs that retain the mCherry fluorescence, while GFP fluorescence is quenched. Rainbow pseudo-colored GFP channel also shown. **Right:** Pixel intensity plot for dashed line. Arrow indicates the vesicle. **e**, Number of mCherry^+^/GFP^-^ VDIMs in experiments as in (**d**) (n= 330 vesicles, 26 cells, 3 experiments). **f**, CLEM analysis of cells expressing LAMP1-GFP (green) and mito-BFP (cyan), labeled with Mitotracker (magenta). Scale bars, 10μm. **Bottom**: Higher magnifications of single-z plane from indicated regions. (i-ii) Lysosome localized VDIMs and presence of membrane whorls. (iii) Lysosome devoid of mitotracker-labeled membrane (arrowheads). Scale bars, 200nm. **g**, Live-cell imaging sequence showing VDIM formation (arrowheads) in cells expressing mito-BFP (blue). Lysosomes were labeled with dextran (green) and mitochondria with mitotracker (magenta). **h**, Mean intensity of mitotracker and mito-BFP in the lysosome over time from (**g**). **i, Top**: Representative data from two experiments showing the percentage of VDIMs forming at mitochondrial midpoint or the periphery in experiments as in (**g**) (n= 52 events, 10 cells). **Bottom:** Schematic illustrating that VDIM formation does not occur at preferential sites along the length of the mitochondria. **j**,**k**, Number of VDIMs in cells with impaired lysosomes. Cells were treated with (**j**) bafilomycin A1 (BafA1) (n= 60 cells, 3 experiments) or (**k**) chloroquine (CQ) (n= 60 cells, 3 experiments). Data shown are mean±SEM shown as large circles and individual data points from corresponding experiments shown in the same colors. Statistical analysis was performed using two-tailed Student’s unpaired t-test. *P* values calculated are shown. Unless stated otherwise, scale bars: 3μm.

Removal of damaged mtDNA is crucial for maintaining mitochondrial homeostasis and several pathways exist to eliminate damage DNA from the mitochondria, including removal via BAX/BAK pores^18^, VDAC pores^19,20^, MDVs^21^ and endosomal trafficking^22^. Given the association between mitochondrial nucleoids and the IMM, mtDNA is particularly sensitive to oxidative stress. Therefore, we considered that VDIM formation might represent a mechanism for mtDNA removal. Although, a proportion of VDIMs contained mtDNA (Fig. 1m-r) and labeling for 8-OHdG revealed that VDIMs contained oxidized DNA (Extended Data Fig. 5g-h). VDIM formation was not impaired in 143b rho0 cells that lack mtDNA^23^ (Extended Data Fig. 5i-j) indicating oxidized mtDNA was not the trigger for their formation. Since our findings suggested that the formation of VDIM occurs as a QC mechanism for IMM removal, we investigated whether VDIMs were destined for lysosomal degradation. As shown in Fig. 3a-b (Extended Data Fig. 6a, Supplementary Video1), VDIMs were localized inside compartments labeled with the lysosomal marker LAMP1, indicating their delivery to the lysosomes. Next, we generated a fluorescent reporter for the IMM localized protein, MCU1 (MCU-GFP-mCherry), where sensitivity of GFP to low pH can be used to monitor the lysosomal localization of the protein of interest (Fig. 3c). As shown in Fig. 3d and Extended Data Fig. 6b, the probe localized to the mitochondria along with TOM20 and mitotracker. However, VDIMs lacked GFP but remained mCherry positive, demonstrating the probe’s localization in an acidic compartment (Fig. 3d-e).

**ED Fig. 7:**
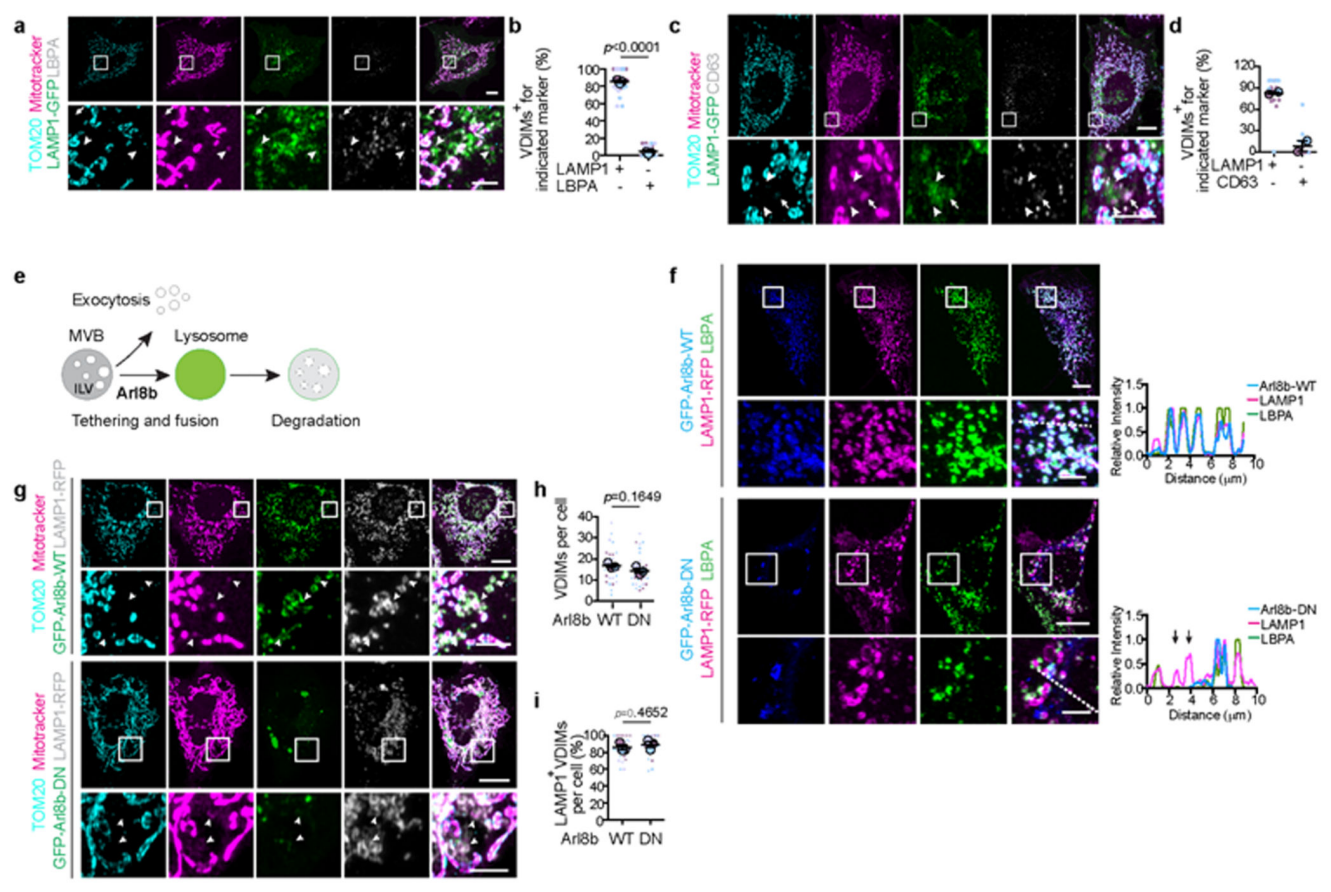
VDIMs are not multivesicular bodies (MVB) **a**, Representative images showing mitotracker (magenta), TOM20 (cyan), LBPA (grey) in cells expressing LAMP1-GFP (green). **Bottom**: Higher magnification of indicated regions. Arrowheads indicate VDIMs lacking LBPA. Arrow indicates VDIM positive for LBPA. **b**, Number of VDIMs positive for LAMP1 or LBPA from experiments as in (**a**) (n=243 vesicles, 30 cells, 3 experiments). **c**, Representative images showing mitotracker (magenta), TOM20 (cyan), CD63 (grey) in cells expressing LAMP1-GFP (green). **Bottom**: Higher magnification of indicated regions. Arrowheads indicate VDIMs lacking CD63. Arrow indicates VDIM positive for CD63. **d**, Number of VDIMs positive for LAMP1 or CD63 from experiments as in (**c**) (n=167 vesicles, 17 cells, 2 experiments). **e**, Schematic illustrating lysosome and MVB fusion regulated by Arl8b GTPase. **f**, Localization of Lamp1 (magenta) and LBPA (green) in cells expressing GFP-Arl8b-WT (blue) or GFP-Arl8b-DN (blue). **Right**: Pixel intensity plots for dashed line. Arrows indicate LBPA negative lysosomes in cells expressing GFP-Arl8b-DN. **g**, VDIM formation in cells expressing GFP-Arl8b-WT (green) or GFP-Arl8b-DN (green). Higher magnification of indicated regions are shown where arrowheads indicate the VDIM. **h**, Number of VDIMs in cells expressing Arl8b-WT or Arl8b-DN in experiments as in (g) (n= 30 cells, 3 experiments). **i**, Number of LAMP1 positive VDIMs in cells expressing Arl8b-WT or Arl8b-DN in experiments as in (**g**) (n=30 cells, 3 experiments). Data shown are mean±SEM. Statistical significance was calculated using two-tailed Student’s unpaired t-test. *P* values calculated are indicated. Scale bars: main panels 10μm, magnified panels 3μm.

To confirm the lysosomal delivery of VDIMs, we performed correlative light and electron microscopy (CLEM), which revealed the presence of mitotracker^+^/matrix^-^ vesicles within lysosomes (Fig. 3f). These appeared as membrane whorls inside lysosomes in CLEM analysis. Of note, lysosomes devoid of mitotracker lacked these luminal membranes (Fig. 3f, Extended Data Fig. 6c, Supplementary Video2). Endocytic organelles, MVBs (multivesicular bodies) are characterized by the presence of intraluminal vesicles and membrane whorls that are generated from the invagination and budding of their limiting membrane^24^. To confirm that VDIMs were indeed lysosomal and not MVBs, we assessed their localization with MVB markers. In contrast to MVBs ^24^, CD63 and LBPA failed to localize with the VDIMs (Extended Data Fig.7a-d). In addition, VDIM formation did not depend on Arlb8 GTPase, which is required for lysosome-MVB fusion^25^ (Extended Data Fig.7e-i), further differentiating VDIM containing lysosomes from MVBs.

Finally, we directly visualized the delivery of VDIMs to lysosomes by performing live-cell imaging of cells expressing mito-BFP (matrix), where mitochondria were also labeled with mitotracker, and lysosomes labeled with fluorescent dextran. Mitotracker^+^/matrix^-^ vesicles pinched off from the mitochondria and were directly delivered into the dextran-labeled lysosomes closely apposed to the mitochondrial surface (Fig. 3g, Extended Data Fig. 6d, Supplementary Videos 3-4). This transfer of mitotracker^+^ vesicles was evident by the concomitant increase in the mitotracker intensity inside the lysosome (Fig. 3h). Notably, sites of VDIM formation showed no preference for the midzone or the periphery along a particular mitochondrion (Fig. 3i). This contrasts with the preferential peripheral fission observed for mitochondria that are delivered to lysosomes for degradation^26^. Similarly, if VDIMs represented remnants from partial proteolysis of mitochondria, inhibiting lysosomal function would cause a decrease in the number of VDIMs. However, treatment with bafilomycinA1 (BafA1) or chloroquine (CQ) to inhibit lysosomal acidification and function caused an increase in the number of VDIMs (Fig. 3j-k, Extended Data Fig. 6e-f). These findings were not limited to TOM20, as Omp25, PDH or SOD2 localization with VDIMs was not altered in BafA1 treated cells (Extended data Fig. 6g-l). Collectively, these data confirmed that VDIMs were delivered to lysosomes for degradation and suggest that VDIM formation could represent a novel intramitochondrial QC pathway to respond to localized IMM damage.

## VDIMs form by mitochondria-lysosome crosstalk

**ED Fig. 8:**
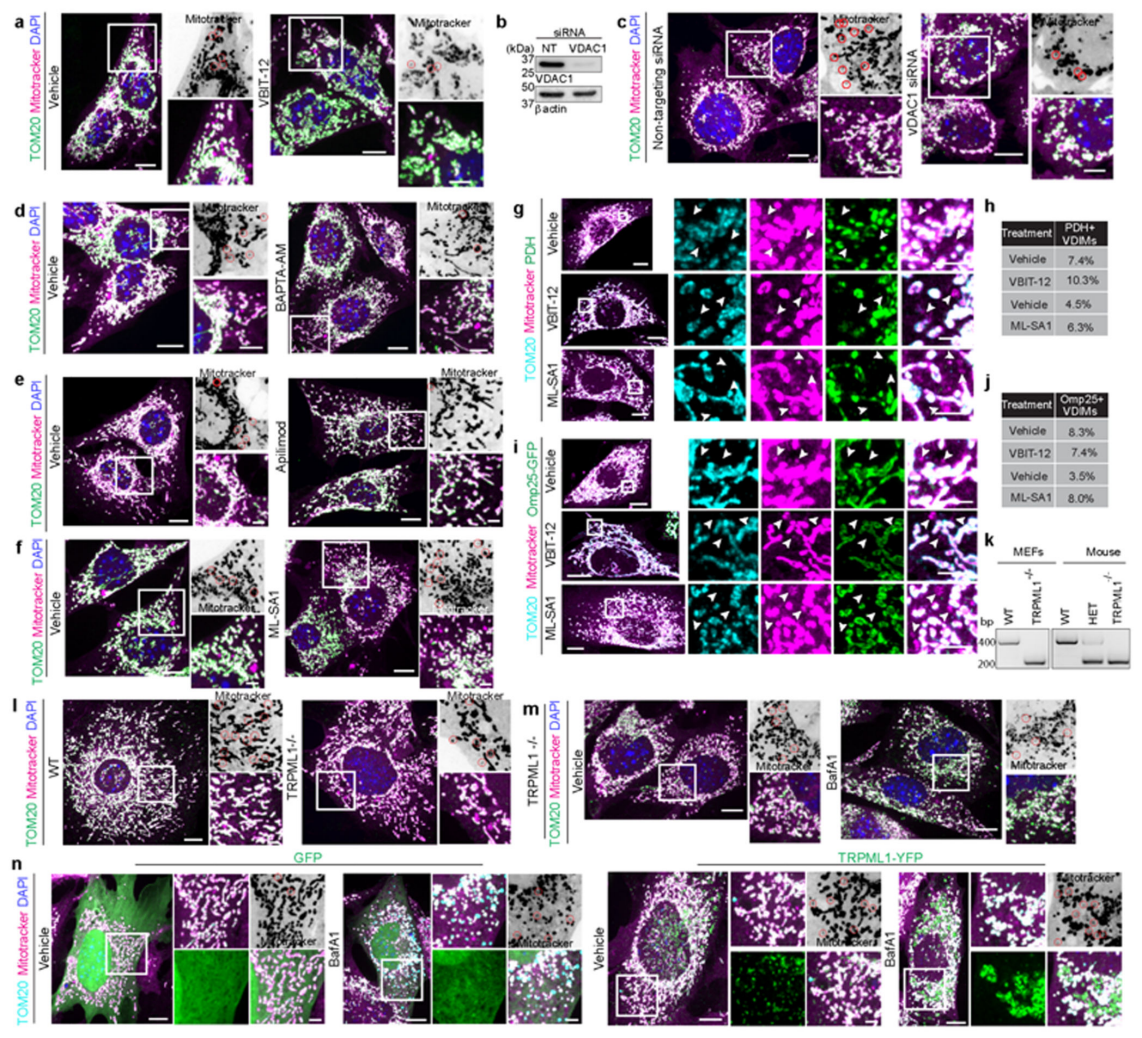
VDAC1 and TRPML1 mediate VDIM formation **a**, VDIM formation in cells treated with vehicle or VBIT-12. **b**, Representative western blot showing the efficiency of VDAC1 knockdown (n=4 experiments). **c**, VDIM formation in cells treated with indicated siRNA. **d**, VDIM formation in cells treated with BAPTA-AM. **e**, VDIM formation in cells treated with apilimod. **f**, VDIM formation in cells treated with ML-SA1. **g**, Mitotracker (magenta), TOM20 (cyan) and PDH (green) localization in cells treated with VBIT-12 or ML-SA1. Arrowheads indicate the VDIMs. **h**, Percentage of mitotracker^+^/TOM20^-^ VDIMs positive for PDH in experiments as in (**g**) (n=293 vesicles for vehicle, 535 vesicles for ML-SA1, 30 cells, 3 experiments for ML-SA1; n=342 vesicles for vehicle, 302 vesicles for VBIT-12, 30 cells, 3 experiments). **i**, Mitotracker (magenta), TOM20 (cyan) in cells expressing Omp25-GFP (green) treated with VBIT-12 or ML-SA1. Arrowheads indicate the VDIMs. **j**, Percentage of mitotracker^+^/TOM20^-^ VDIMs positive for Omp25 in experiments as in (**i**). **k**, Validation of gene knockout in TRPML1 ^**-/-**^ MEFs. MLIV gene was amplified from WT and TRPML1 ^**-/-**^ MEFs and from DNA extracted from ear-notches of WT, KO and heterozygous (het) mice (n=1). **l**, Representative confocal micrographs showing VDIM formation in WT and TRPML1^**-/-**^ MEFs. **m**, VDIM formation in TRPML1^**-/-**^ MEFs treated with BafA1. **n**, Effect of TRPML1 re-expression on VDIM formation in TRPML1^**-/-**^MEFs. TRPML1^**-/-**^ cells transiently transfected with GFP or TRPML1-YFP were treated with vehicle or BafA1. Data shown are means from 3 experiments. Representative spinning disc confocal micrographs are shown in (**a, c-f, l-n**). For all fluorescence images, higher magnifications of indicated regions are shown to the right. Red circles on the inverted fluorescence micrographs for the mitotracker channel indicate the VDIMs. Gel source data for (**b**) are provided in Supplementary Fig.2. Scale bars: main panels 10μm, magnified panels 3μm.

**Fig. 4:**
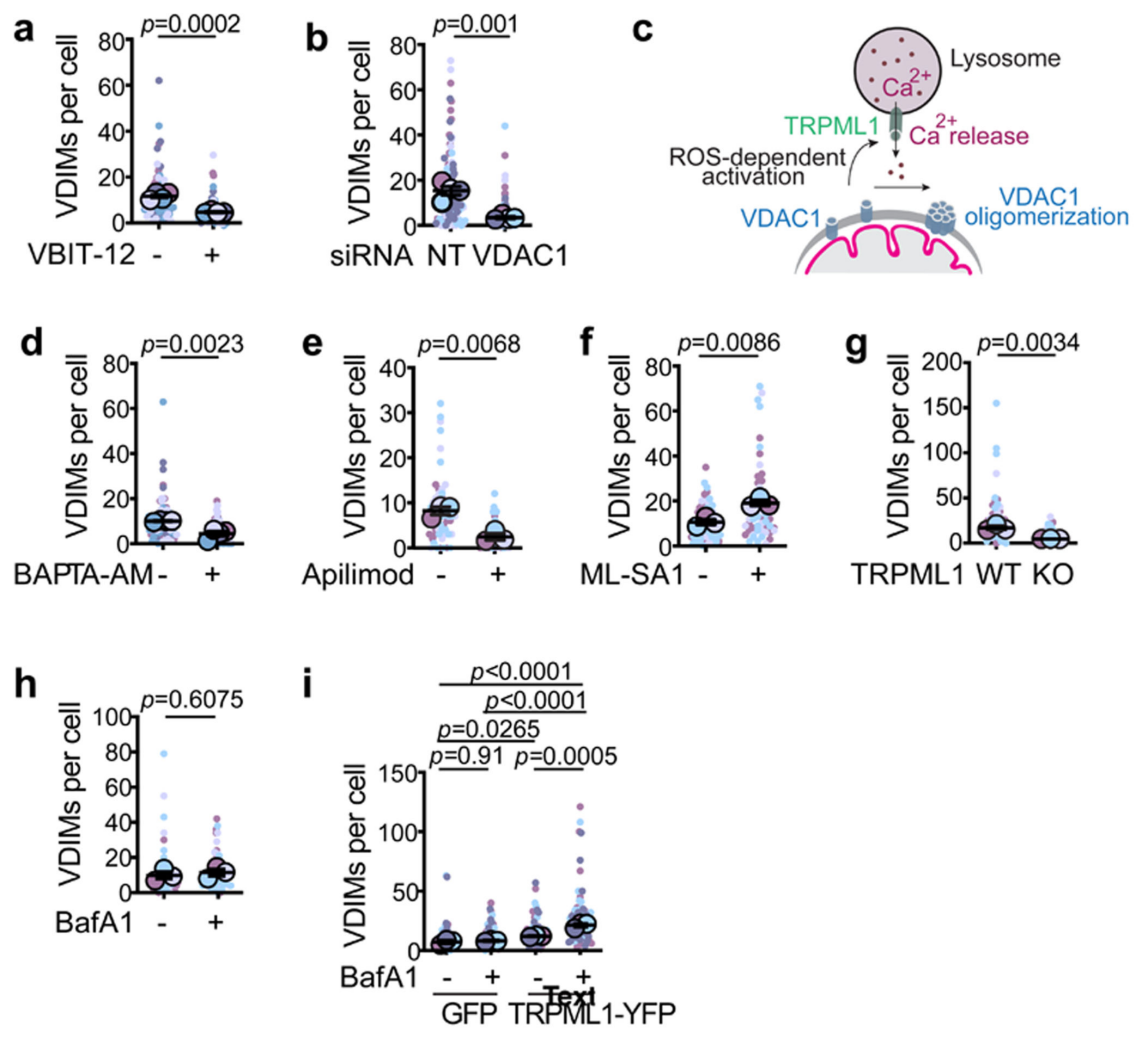
VDAC1 and lysosomal Ca^2+^ channel TRMPL1 mediate VDIM formation **a**, Number of VDIMs in cells treated with VBIT-12 (n= 81 cells for vehicle, 83 for VBIT-12, 4 experiments). **b**, Number of VDIMs in cells treated with scrambled (NT) or VDAC1 siRNA (n= 36 cells, 4 experiments). **c**, Schematic illustrating the mitochondria-lysosome crosstalk mediated by VDAC1 and TRPML1. **d**, Number of VDIMs in cells treated with vehicle (-) or BAPTA-AM (n= 80 cells, 4 experiments). **e**,**f**, Effect of TRPML1 activity on VDIM formation. Cells were treated with (**e**) Apilimod (n= 60 cells, 3 experiments) or (**f**) ML-SA1 (n= 60 cells, 3 experiments). **g-i**, Effect of TRPML1 on VDIM formation. **g**, Number of VDIMs formed in TRPML1^-/-^ (KO) and littermate WT MEFs (n= 55 cells, 3 experiments). **h**, VDIM formation in TRPML1 KO MEFs treated with vehicle (-) or BafA1 (n= 62 cells, 3 experiments). **i**, Rescue of VDIM formation in TRPML1 KO MEFs re-expressing TRPML-1. TRPML1 KO MEFs were transiently transfected with GFP or TRPML1-YFP. Cells were treated with vehicle (-) or BafA1 (+) to allow VDIMs to accumulate (n=59 for GFP+vehicle, 62 for GFP+BafA1, 60 for TRPML1-YFP+vehicle, 60 for TRPML1-YFP+BafA1, 3 experiments). Data shown are mean±SEM shown as large circles and individual data points from corresponding experiments shown in the same colors. Statistical analysis was performed using two-tailed Student’s unpaired t-test (**a-b, d-h**) and One-way ANOVA followed by Tukey’s multiple-comparison test (**i**). *P* values calculated are shown.

We next dissected the pathway by which VDIMs were delivered to lysosomes. VDIM formation spared the rest of the mitochondrial filament, suggesting that their formation occurred without rupturing the OMM. The OMM-localized voltage-gated anion channel (VDAC) can oligomerize to form large pores causing the release of mitochondria DNA^20^. These pores, which can consist of up to 20 monomers^27,28^, form in response to oxidative stress and increases in cytosolic calcium (Ca^2+^)^29^. Therefore, we investigated if VDAC1 was needed for VDIM formation. Treatment with VDAC inhibitor VBIT-12^30^ or silencing VDAC1 expression precluded VDIM formation (Fig. 4a-b, Extended Data Fig. 8a-c), supporting a role of VDAC1 in VDIM formation. Interestingly, VDAC interacts with the lysosomal Ca^2+^ channel TRPML1 (transient receptor potential mucolipin1)^31^ which responds to mitochondrial ROS by releasing Ca^2+ 32^, which in turn can regulate mitochondrial function^33^. Given the proximity between mitochondria and lysosome during VDIM formation, we explored the role of TRPML1-Ca^2+^-VDAC axis in their formation (Fig. 4c). Treatment with cell permeable Ca^2+^ chelator BAPTA-AM caused a significant reduction in VDIM formation (Fig. 4d, Extended Data Fig. 8d). Similarly, impairing TRPML1 activity by apilimod^34^ reduced, while treatment with its agonist ML-SA1^35^ induced VDIM formation (Fig. 4e-f, Extended Data Fig. 8e-f). Of note, targeting VDAC or TRPML1 did not alter the localization of Omp25 or PDH with the VDIMs (Extended data Fig. 8g-j).

We further confirmed the involvement of TRPML1-dependent Ca^2+^ release in VDIM formation by generating TRPML1^-/-^ and littermate WT MEFs (Extended Data Fig. 8k). At steady state VDIM formation was inhibited in TRPML1^-/-^ MEFs (Fig. 4g, Extended Data Fig. 8l). Furthermore, compared to WT cells (Fig. 3j), TRPML1^-/-^ cells failed to accumulate VDIMs in the presence of BafA1 (Fig. 4h, Extended Data Fig. 8m). This defect in VDIM formation was rescued upon re-expression of TRPML1 in TRPML1^-/-^ cells (Fig. 4i, Extended Data Fig. 8n). Taken together, these results illustrate that the inter-organelle communication between mitochondria and lysosomes facilitates VDIM formation.

## VDIMs form by ESCRT-mediated microautophagy

**ED Fig. 9:**
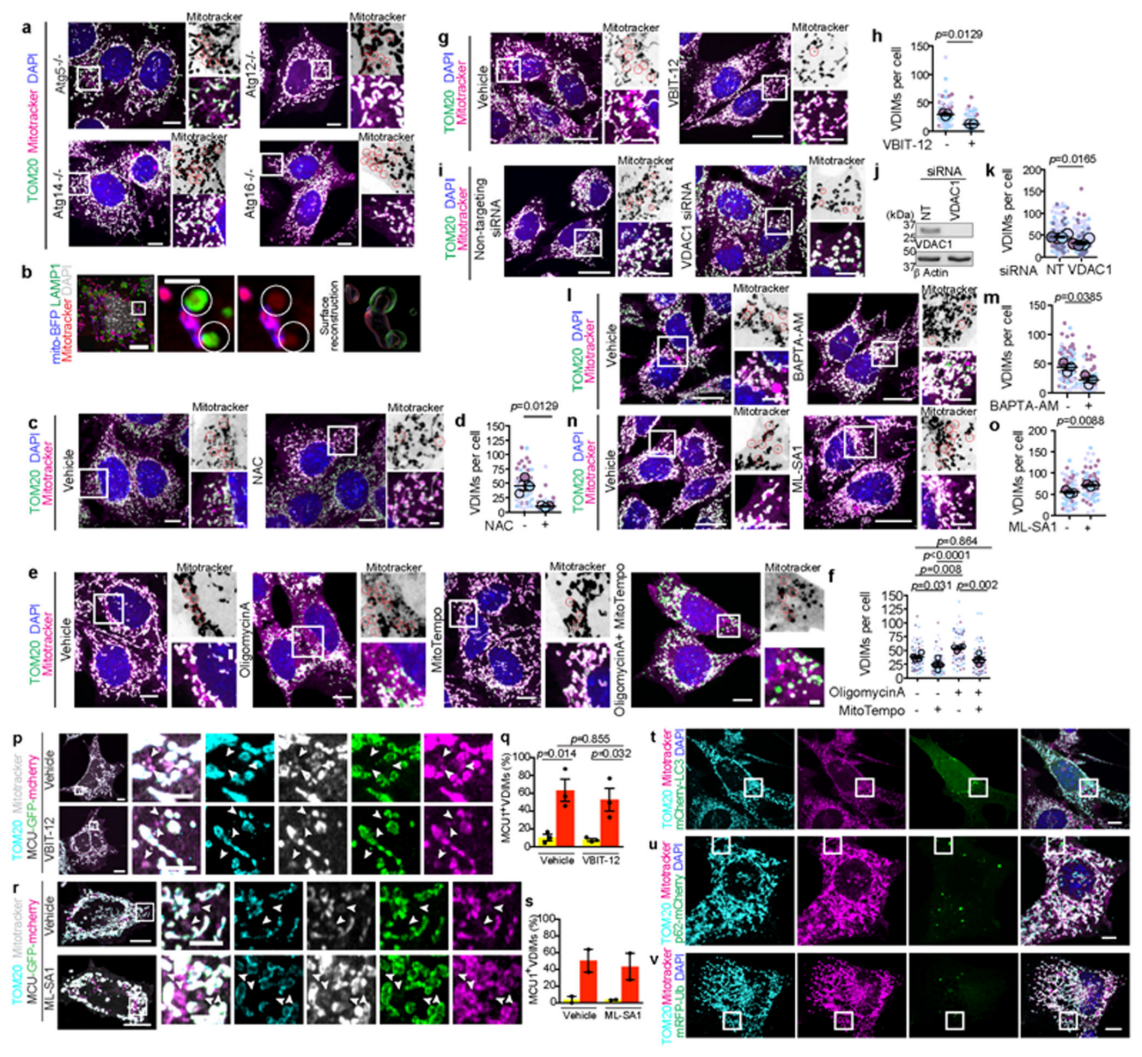
VDIMs form independently of macroautophagy **a**, VDIM formation in MEFs lacking Atg5, Atg12, Atg14 and Atg16. **Right**: Higher magnification of indicated regions. **b**, VDIM formation in Atg5-/- MEFs. Representative confocal micrographs showing VDIM pinching off and being directly sorted into a lysosome. **Right:**3D-reconstruction of indicated region. **c**, Effect of scavenging ROS on VDIM formation. Cells were treated with vehicle (-) or NAC (+). **d**, Number of VDIMs in cells in experiments as in (**c**) (n=63 cells, 3 experiments). **e**, Effect of oxidative stress on VDIM formation. Cells were treated with vehicle, oligomycinA, mitoTempo or oligomycinA and mitoTempo together. **f**, Number of VDIMs in cells in experiments as in (e) (n= 75 cells for vehicle, 64 cells for oligomycinA, 73 cells for mitoTempo, 66 for oligomycinA+mitoTempo, 4 experiments). **g**, Effect of VDAC inhibition on VDIM formation. **h**, Number of VDIMs in cells treated with VBIT-12 in experiments as in (**g**) (n=75 cells, 3 experiments). **i**, Representative images showing VDIM formation in cells treated with indicated siRNA. **j**, Representative western blot showing the efficiency of VDAC1 knockdown (n=4 experiments). **k**, Number of VDIMs in VDAC1 depleted cells in experiments as in (**i**) (n=136 cell for NT, 127 for VDAC siRNA, 4 experiments). **l**, Representative images showing the effect of BAPTA-AM on VDIM formation. **m**, Number of VDIMs in cells treated with BAPTA-AM in experiments as in (**l**) (n= 87 cells for vehicle, 67 cells for BAPTA-AM, 3 experiments). **n**, Representative images showing VDIM formation in cells treated with ML-SA1. **o**, Number of VDIMs in cells treated with ML-SA1 in experiments as in (**n**) (n=66 cells for vehicle, 69 cells for ML-SA1, 3 experiments). **p**, Effect of VDAC1 inhibition on MCU-GFP-mCherry positive VDIMs. Cells expressing MCU-GFP-mCherry were treated with VBIT-12. **q**, Number of VDIMs positive for GFP+mCherry (yellow) and mCherry (red), indicating lysosomal quenching of GFP from experiments as in (**p**) (n= 412 vesicles for vehicle, 336 vesicles for VBIT-12, 30 cells, 3 experiments). **r**, Effect of TRPML1 activation by ML-SA1 on MCU-GFP-mCherry positive VDIMs. **s**, Number of VDIMs positive for GFP+mCherry (yellow) and mCherry (red), indicating lysosomal quenching of GFP from experiments as in (**r**) (n=229 vesicles for vehicle, 194 vesicles for ML-SA1, 20 cells, 2 experiments). **t-v**, Localization of TOM20 (cyan) and mitotracker (magenta) with indicated autophagy markers. Cells were transfected with (**t**) mCherry-LC3 (green), (**u**) p62-mCherry (green), (**v**) mRFP-Ub (green). Higher magnifications of indicated regions are shown in Fig.5e. Representative spinning disc confocal micrographs are shown in (**a, c, e, g, i, l, n, p, r**) where higher magnification of indicated regions are shown to the right. Red circles in inverted micrograph for mitotracker indicate the VDIMs. Data shown are mean±SEM shown as large circles and individual data points from corresponding experiments shown in the same colors. Statistical significance was calculated using two-tailed Student’s unpaired t-test in (**d, h, k, m, o**), and One-way ANOVA followed by Tukey’s multiple-comparison test in (**f, q, s**). *P* values calculated are indicated. Gel source data for (**j**) are provided in Supplementary Fig.1. Scale bars: main panels 10μm, magnified panels 3μm.

**ED Fig. 10:**
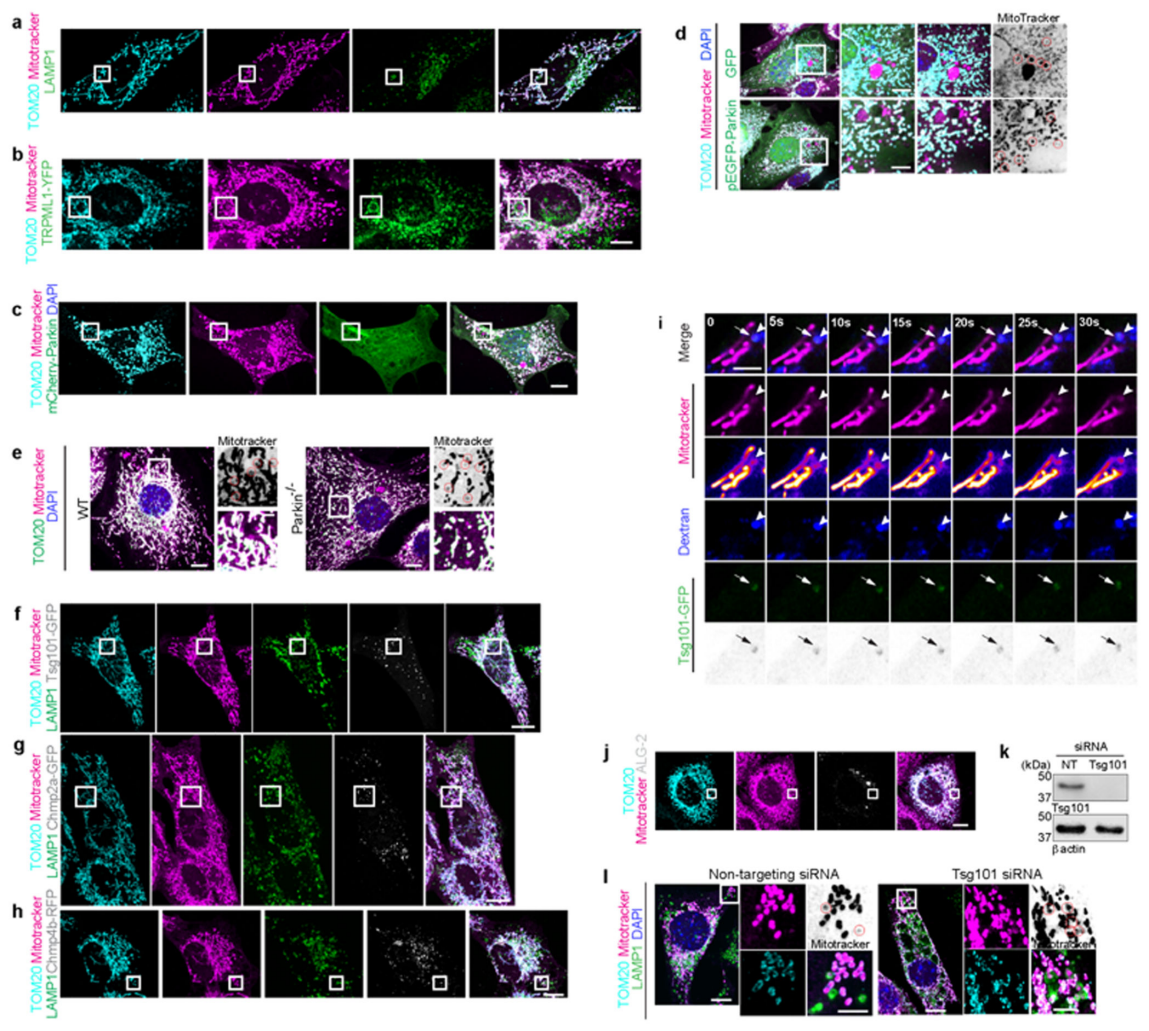
VDIMs form by ESCRT-mediated, microautophagy-like process **a**, Representative images showing TOM20 (cyan), mitotracker (magenta) and LAMP1 (green). Higher magnifications of indicated regions are shown in Fig.5g. **b**, Representative images showing localization of TOM20 (cyan), mitotracker (magenta) and TRPML1 (green). Cells were transiently transfected with TRPML1-YFP. Higher magnifications of indicated regions are shown in Fig.5h. **c**, Localization of TOM20 (cyan) and mitotracker (magenta) in cells expressing mCherry-Parkin. Higher magnification of indicated region is shown in Fig.5i. **d**, Effect of Parkin overexpression on VDIM formation. Cells were transiently transfected with (top) GFP or (bottom) pEGFP-Parkin. Red circles in inverted micrographs for mitotracker indicate VDIMs. **e**, Representative images showing VDIM formation in Parkin-/- MEFs compared to WT controls. Circles in the inverted mitotracker micrograph indicate the VDIMs. **f-h**, Representative images showing localization of TOM20 (cyan), mitotracker (magenta) and LAMP1 (green) in cells transiently transfected with (**f**) Tsg101-GFP (grey), (**g**) Chmp2a-GFP (grey) or (**h**) Chmp4b-RFP (grey). Higher magnifications of indicated regions are shown in Fig.6a-c. **i**, Live-cell imaging sequence showing recruitment of Tsg101 (green) at sites of VDIM scission. Images were acquired every 5 sec. Arrowheads indicate the VDIMs and arrows indicate the Tsg101 puncta. **j**, Representative images showing mitotracker (magenta), TOM20 (cyan) and ALG-2 (grey). Higher magnifications of the indicated regions are shown in Fig. 6g. **k**, Representative western blot showing the efficiency of Tsg101 depletion compared to non-targeting (NT) controls. **l**, Representative images showing VDIM formation in cells treated with Tsg101 siRNA compared to NT controls. **Right**: Higher magnification of indicated regions. Red circles in inverted micrograph for mitotracker indicate the VDIMs. All data shown are representative from three independent experiments. **d**,**e**, Representative spinning disc confocal images are shown. Gel source data for (**k**) are provided in Supplementary Fig.1. Scale bars: main panels 10μm, magnified panels 3μm.

**Fig 5:**
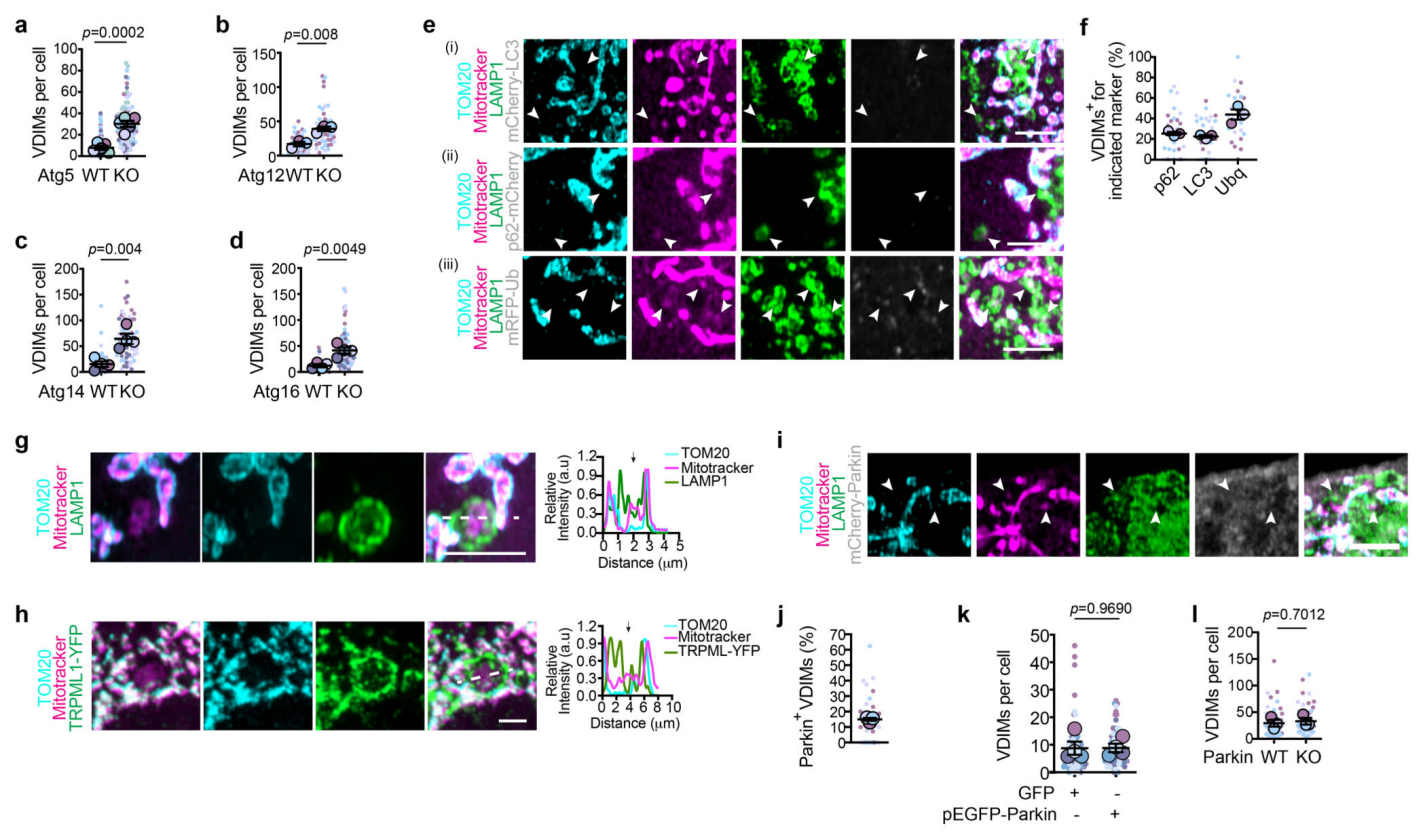
VDIMs form by engulfment of IMM by lysosomes in a microautophagy-like process **a-d**, Number of VDIMs in (**a**) Atg5^-/-^ (KO) (n= 101 WT, 98 KO cells, 5 experiments), (**b**) Atg12^-/-^ (KO) (n= 40 WT, 46 KO cells, 3 experiments), (**c**) Atg14^-/-^ (KO) (n= 61 WT, 91 KO cells, 4 experiments), and (**d**) Atg16^-/-^ (KO) (n= 73 WT, 72 KO cells, 4 experiments) MEFs compared to MEFs from littermate WT controls. **e**, Localization of VDIMs and indicated autophagy markers. Mitochondria in cells expressing (i) mCherry-LC3 (grey) (n=264 vesicles, 29 cells, 3 experiments), (ii) p62-mCherry (grey) (n=285 vesicles, 30 cells, 3 experiments) or (iii) mRFP-Ub (grey) (n=264 vesicles, 30 cells, 3 experiments) labeled with mitotracker (magenta) and TOM20 (cyan) to identify the VDIMs (arrowheads). **f**, Percentage of VDIMs positive for indicated autophagy markers in experiments as in (e) (n=331 vesicles, 30 cells, 3 experiments for p62; n= 261 vesicles, 30 cells, 3 experiments for LC3; n= 394 vesicles, 31 cells, 3 experiments for Ubq). **g**,**h**, Lysosome membrane (**g**) LAMP1 or (**h**) TRPML1 vesicles inside the lumens of lysosomes that contain the VDIMs. **Right:** Pixel intensity plot for dashed line. Arrows indicate the VDIMs. **i**, Lack of Parkin localization with VDIMs (arrowheads) in cells expressing mCherry-Parkin (grey). **j**, Percentage of VDIMs positive for Parkin in experiments as in (i) (n=240 vesicles, 28 cells, 3 experiments). **k**, Number of VDIMs in cells overexpressing GFP or pEGFP-Parkin (n=80 cells, 4 experiments). **l**, Number of VDIMs in Parkin^-/-^ (KO) MEFs compared to littermate WT control MEFs (n=57 cells, 3 experiments). Data shown are mean±SEM shown as large circles and individual data points from corresponding experiments shown in the same colors. Statistical analysis was performed using two-tailed Student’s unpaired t-test (**a**-**d**,**k**-**l**). *P* values calculated are shown. Scale bars: 3μm.

Delivery of cargo to lysosomes can occur indirectly by fusion of autophagosomes with lysosomes during macroautophagy^36^. Alternatively, lysosomes directly take up cargo by recognition of KFERQ-like motif bearing cytosolic proteins during chaperone-mediated autophagy^37^, or via microautophagy^38^, where invagination of lysosomal membrane sequesters cargo, followed by membrane scission leading to cargo internalization and formation of intraluminal vesicles positive for lysosomal membrane proteins.^39–42^. VDIM formation was not abrogated in cells lacking the core autophagy machinery components Atg5, Atg12, Atg14 and Atg16L1^43^ (Fig. 5a-d, Extended Data Fig. 9a), and the vesicles were generated by the same mechanism in the absence of Atg5 (Extended Data Fig. 9b-s). Additionally, the canonical autophagy markers, p62 and LC3B^43,44^ failed to localize with VDIMs (Fig. 5e-f, Extended Data Fig. 9t-u). Therefore, we ruled out the role of macroautophagy in VIDM formation. Notably, the absence of macroautophagy led to a greater number of VDIMs. Interestingly, 40% of the VDIMs were positive for ubiquitin (Fig. 5e-f, Extended Data Fig. 9v). Recognition of ubiquitinated cargo is important for lysosomal microautophagy,^45^ and as would be expected for membrane invagination during microautophagy, lysosomal transmembrane proteins were present in the lumens of lysosomes that contained VDIMs (Fig. 5g-h, Extended Data Fig. 10 a-b, Supplementary Video5), altogether supporting a role of a microautophagy-like process in VDIM formation. The E3 ligase Parkin is the most well-studied regulator of mitochondrial ubiquitination and mitophagy ^46^. However, VDIMs lacked staining for Parkin (Fig. 5i-j, Extended Data Fig. 10c), and neither the over-expression nor the deletion of Parkin altered their formation (Fig. 5k-l, Extended Data Fig. 10d-e), demonstrating that VDIM formation occurred independently of Parkin.

**Fig 6:**
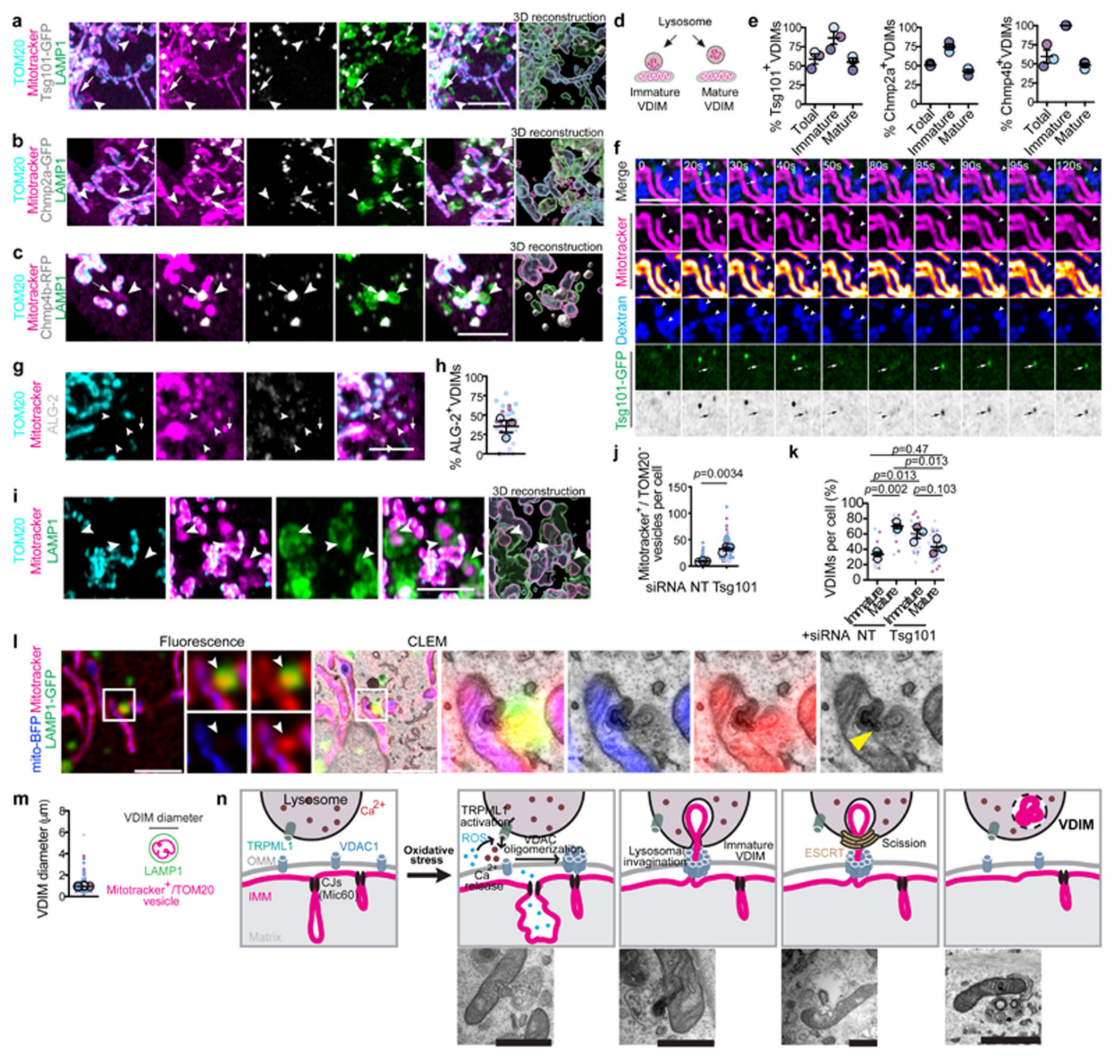
The ESCRT complex mediates VDIM formation **a**, Tsg101-GFP, **b**, Chmp2a-GFP, or **c**, Chmp4b-RFP (grey) localization with VDIMs (arrowheads). Arrows indicate ESCRT puncta. **Right**: 3D-reconstruction. **d**, Schematic illustrating VDIM stages observed in fixed-cell imaging. **e**, Percentage of immature and mature VDIMs positive for indicated proteins in experiments as in (a-c) (n=31 cells for Tsg101, 30 cells for Chmp2a, 30 cells for Chmp4b, 3 experiments). **f**, Live-cell imaging sequence showing Tsg101 recruitment at sites of VDIM scission. Images were acquired every 5 sec. Arrowheads indicate the VDIMs and arrows indicate the Tsg101 puncta (n=25 events). **g**, ALG-2 (grey) positive VDIMs (arrowheads). Arrow indicates an ALG-2 negative VDIM. **h**, Percentage of VDIMs positive for ALG-2 from experiments as in (g) (n=408 vesicles, 29 cells, 3 experiments). **i**, Representative images showing impaired vesicle scission (arrowheads) after Tsg101 depletion. **Right:** 3D-reconstruction. **j**, Number of mitotracker^+^/Tom20^-^ vesicles after Tsg101 depletion (n=71 cells, 3 experiments). **k**, Percentage of immature and mature VDIMs after Tsg101 depletion from (j). **l**, CLEM analysis of cell expressing LAMP1-GFP (green) and mito-BFP (cyan) after Tsg101 depletion. Mitotracker (magenta). Arrowhead indicates the IMM herniating into the lysosome. **m**, Size distribution of VDIMs from airyscan images (n=548 vesicles, 42 cells, 4 experiments). **Right**: Schematic illustrating the IMM encapsulated by a lysosome, forming a VDIM. **n, Top**: Schematic illustrating the proposed mechanism of VDIM formation. **Bottom**: EM micrographs of Tsg101 depleted cells from (l), illustrating the different stages of VDIM formation. All data shown are from at least three independent experiments with mean±SEM shown as large circles and individual data points from corresponding experiments shown in the same colors. Statistical significance was calculated using two-tailed Student’s unpaired t-test for (**j**) and One-way ANOVA followed by Tukey’s multiple-comparison test for (**k**). *P* values calculated are shown. Scale bars, 3μm (a-c, f-g,i), and 10μm for fluorescence and 200nm for EM image in (l).

Our findings indicated the presence of a mechanism of lysosomal sequestering of IMM and membrane scission. Therefore, we examined whether the ESCRT machinery, which participates in membrane scission during microautophagy^47^ was involved. In fixed cells, ESCRT complex proteins could be detected at potential sites of VDIM scission (Fig. 6a-e, Extended Data Fig. 10f-h, Supplementary Videos 6-8), where ‘immature’ VDIMs transition to ‘mature’ VDIMs, fully internalized by the lysosomes, as well as on ‘mature’ VDIMs. Notably, greater percentage of immature VDIMs were positive for the ESCRT proteins, indicative of their role in mediating membrane scission for VDIM formation (Fig. 6e). Furthermore, live-cell imaging of cells expressing Tsg101-GFP, labeled with mitotracker and fluorescent dextran (lysosomes), revealed the recruitment of Tsg101 at the sites where mitotracker^+^ vesicles pinched off and were engulfed by the lysosomes (Fig. 6f, Extended Data Fig. 10i, Supplementary Video 9-10), thus indicating a role for ESCRTs in VDIM formation. The recruitment of ESCRT machinery required for membrane scission at the site of damaged plasma membrane and lysosomes is triggered by calcium sensing by ALG-2 (apoptosis-linked gene 2)^48^. Considering the role of calcium in VDIM formation, we wondered whether ALG-2 similarly regulated ESCRT recruitment for VDIM formation. Indeed, in support for a role for ALG-2 in ESCRT recruitment during VDIM formation, 35.4% of VDIMs were positive for ALG-2 (Fig. 6g-h, Extended Data Fig. 10j). If the ESCRT machinery facilitates membrane scission needed for the final step in VDIM formation, we surmised that its absence would result in stalled microautophagy, leading to partially formed VDIMs. Consistent with this, in cells where expression of ESCRT protein Tsg101 was depleted, we observed mitotracker^+^/TOM20^-^ vesicles being internalized by lysosomes that appeared to remain attached to the mitochondrial filaments, indicating impaired scission (Fig. 6i, Extended Data Fig. 10l, Supplementary Video 11). This failure in scission required for VDIM formation, and the subsequent lack of lysosomal degradation of the IMM fragments, resulted in an increase in the number of ‘immature’ VDIMs present in these cells (Fig. 6j-k). CLEM analysis of Tsg101 depleted cells clearly showed the IMM herniating into a lysosome, resembling an ‘immature’ VDIM (Fig. 6l). These findings, along with the observations that VDIMs were always present inside lysosomes (Fig. 3b) suggested that lysosomes were required for VDIM formation. Collectively, these data demonstrated that a microautophagy-like process, mediated by the ESCRT machinery leads to formation of VDIMs which are IMM sections that are encapsulated by lysosomes, and range in size from 0.1-5.6μm (Fig. 6m). Alterations in cristae size, shape and integrity are associated with several diseases including cancer and neurodegeneration^49^. However, the contribution of aberrant IMM dynamics in these processes is unknown. Here we provide a mechanistic description of a newpathway of intramitochondrial QC, facilitated by IMM compartmentalization and mediated by IMM remodeling. We propose that ROS release by damaged cristae locally activates TRPML1 channels on lysosomes in proximity to the mitochondria leading to VDAC1 oligomerization. This oligomerization creates a pore in the OMM through which the damaged IMM fragment herniates and is directly taken up by the closely apposed lysosome. Given the close association between the IMM and mitochondrial nucleoids, we propose that mtDNA is taken up along with the herniating IMM. Next, ESCRT machinery-mediated membrane scission leads to their lysosomal internalization and the formation of VDIMs, resulting in the piecemeal removal of damaged IMM, while sparing the rest of the organelle (Fig. 6n).

Impairment of VDIM formation could lead to mitochondrial retention of damaged cristae. The consequent accumulation of oxidative damage would contribute to functional impairment of mitochondria (e.g. mtDNA damage, impaired bioenergetics). Therefore, the compartmentalized organization per se could serve as a IMM QC mechanism. In support of this rationale, the IMM protein MTFP1 (mitochondrial fission process 1) has been recently shown to selectively isolate damaged IMM subdomains and nucleoids to facilitate their removal by an autophagy-dependent process^50^. A better understating of the regulation of the dynamic IMM organization, its remodeling and maintenance should lead to identification of potential therapeutic targets for mitochondrial diseases or other pathological processes where mitochondrial homeostasis is disrupted.

## Methods

### Reagents

10-N-nonyl acridine orange (NAO) (Invitrogen, #A1372) was used at 100nM for 2h. MitoCLox (Lumiprobe, #3549) was used at 200nM for 1h. Bafilomycin A1 (Cayman Chemicals, #11038) was used at 50nM for 24h. N-acetyl-cysteine (NAC) (Sigma, #A7250) was used at 5mM for 24h. MitoTempo (Sigma, #SM10737) was used at 50 μM for 24h. Oligomycin (Santa Cruz, #sc201551) was used at 10μM for 24h. Rotenone (Abcam, #ab143145) was used at 10 μM for 24h. VBIT-12 (Cayman Chemicals, #31445) was used at 10μM for 24h. ML-SA1 (Sigma, #SML0627) was used at 20μM for 24h. BAPTA-AM (ThermoFisher, #B1205) was used at 10μM for 24h. MitoTrackerCMXRos (ThermoFisher, #M7512) and MitoTracker DeepRed (ThermoFisher, #M22426) were used at 100nM for 15min. Dextran cascade blue (ThermoFisher, #D1796) and Dextran488 (ThermorFisher, #D22910). All primers used were from integrated DNA technologies (Iowa, USA).

### Plasmids and transfection

mito-BFP was a gift from Gia Voeltz (Addgene plasmid #49151), pDEST47-MCU-GFP was a gift from Vamsi Mootha (Addgene plasmid #31732), LAMP1-GFP was a gift from Ron Vale (Addgene plasmid #16290), TRPML1-YFP was a gift from Craig Montell (Addgene plasmid # 18826), CHMP2A_GFP_N_term was a gift from Daniel Gerlich (Addgene plasmid #31805), pLNCX2-mCherry-CHMP4B was a gift from Sanford Simon (Addgene plasmid #116923). pEGFP-parkin was a gift from Edward Fon (Addgene plasmid #45875), mCherry-Drp1 was a gift from Gia Voeltz (Addgene plasmid # 49152), mCherry-Parkin was a gift from Richard Youle (Addgene plasmid #23956), TFAM-mScarlet was a gift from Stephen Tait (Addgene plasmid #129573). PolG2-tGFP was from OriGene (#RG203462). Tsg101-GFP, described in^51^ was provided by Dr. Sergio Grinstein (The Hospital for Sick Children, Toronto, Canada). GFP-Arl8b, GFP-Arl8b-DN^52^, p62-mCherry^53^ were provided by Dr. John Brummell (Hospital for Sick Children, Toronto, Canada) and have been previously described. mApple-TOM20, mCherry-LC3, SOD2-GFP and GFP-Ubq were generously provided by Dr. Peter Kim (The Hospital for Sick Children, Toronto) and been previously described^54–57^.

MCU-GFP-mCherry was made for this study.

mCherry insert was amplified from mCherry-Drp1 and sub-cloned into pDEST47-MCU-GFP using In-Fusion protocol from TaKaRa Bio (#638954).

Insert F- 5’- GACGAGCTGTACAAGATGGTGAGCAAGGGCGAGG-3’

Insert R- 5’- TTAAACTTATCATTAACTTGTACAGCTCGTCCATGCC-3’

Vector F- 5’- TAATGATAAGTTTAAACGGGGGAGG-3’

Vector R- 5’ CTTGTACAGCTCGTCCATGCC-3’

In all cases cells were analyzed after overnight transfection with indicated plasmids using FuGENE-HD transfection reagent (Promega, #E2311).

### Cell culture

AGS, HeLa, ModeK and Cos-1 were from American Type Culture Collection (ATCC). NCI-H292 cells, originally from ATCC were generously provided by Dr. Mauricio Terebiznik (University of Toronto at Scarborough, Toronto, Canada). Drp1^-/-^ and wild-type littermate control MEFs have been previously defined^58^. Parkin^-/-^ and wild-type littermate control MEFs have been previously described^59^. Atg5^-/-^ and control MEFs were provided by Dr. Noboru Mizushima (University of Tokyo). Atg14^-/-^, Atg16L1^-/-^and corresponding wild-type littermate controls were generously provided by Dr. Tamotsu Yoshimori (Osaka University, Japan) and were previously described^60^. MEFs, HeLa and Cos-1 cells were cultured in Dulbecco’s Modified Eagles Medium (DMEM), 2mM L-glutamine, non-essential amino acids supplemented with 10% fetal-bovine serum (FBS) at 37**°**C with 5% CO_2._ NCI-H292 cells were cultured in RPMI-1640 medium, 2mM L-glutamine supplemented with 10% FBS.AGS cells were cultured in Ham’s F-12 culture medium, 2mM L-glutamine supplemented with 10% FBS. HeLa cells stably expressing Mito-dsRED and EGFP-Parkin were generously provided by Dr. John Brummell (Hospital for Sick Children, Toronto, Canada) and have been previously described^61^. Previously described^62^ 143b ρ0 and control cells, were provided by Dr. Neal Sondheimer (Hospital for Sick Children, Toronto) and were cultured in DMEM supplemented with 10%FBS, pyruvate and 50μg/ml uridine.

### Generation of TRPML1^-/-^ MEFs

*Trpml1* heterozygous mice were obtained from Susan Slaugenhaupt (Massachusetts General Hospital, Harvard Medical School) and bred to obtain *Trpml1*-/- and wild-type littermates. MEFs were generated as described previously^63^. Briefly, embryos were harvested ∼14 days after the appearance of a copulation plug. Tissue was cut into 1-2mm pieces and digested in 0.25% trypsin-EDTA at 37**°**C for 10min, pipetted up and down several times and incubated at 37**°**C for an additional 5min. Tissue suspension was added to MEF culture medium (DMEM+10% FBS/2mM L-glutamine/1X penicillin-streptomycin), larger tissue fragments allowed to settle and supernatant consisting of single cells was transferred to T75 flask. Once confluent, cells were serial-passaged every 5 days for several weeks. Immortalized MEFs were obtained at passage 20.

TRPML1 gene knockout from MEFs was confirmed by PCR using the following primers:

WT Forward: 5’- TGA GGA GAG CCA AGC TCA TT-3’

WT Reverse- 5’- TCA TCT TCC TGC CTC CAT CT-3’

NeoR- 5’- TGG CTG GAC GTA AAC TCC TC-3’

### siRNA knockdowns

Non-targeting siRNA (ON TARGETplus SMARTpool D-001810-10-05), siRNA targeting Tsg101 (L-049922-01-0005), siRNA targeting Vdac1 (L047345-00-0005), siRNA targeting mic60/IMMT (L-046765-01-0005), siRNA targeting Miro1(L-063998-01-0005) and siRNA targeting Snx9 (L-057505-01-0005) were from Dharmacon (Horizon Discovery, Waterbeach, UK). After overnight plating, 25nM of each siRNA was added for 48h. Media was removed and an additional 25nM of each siRNA was added for 24h. Cells were lysed for western blot analysis or fixed in 4% paraformaldehyde for imaging as described below. Knockdowns were performed using Dharmafect transfection reagent (T-2001-01, Dharmacon, Horizon Discovery).

### Depletion of mitochondria

Mitochondria were depleted following the previously described protocol^10^. Briefly, HeLa cells stably expressing mito-dsRed and eGFP-Parkin^61^ were treated with 12.5μM CCCP every 12hr for 72h. Expression of mitochondrial proteins was assessed by immunoblotting and immunofluorescence

### SDS-PAGE and Immunoblotting

At indicated times, cells were lysed in RIPA buffer containing protease inhibitors for 20min on ice. Lysates were denatured with Lammeli sample buffer containing 2-mercaptoethanol, boiled for 10 min and proteins were resolved by SDS-PAGE and transferred to nitrocellulose membranes (BioRad). Membranes were blocked with 5% non-fat milk in TBS-T (Tris-buffered saline with 0.1% Tween-20) for 1h at RT. Membranes were incubated with primary antibody at appropriate dilution in blocking solution overnight at 4**°**C. Membranes were washed 3 times for 10 min each with TBS-T and incubated with secondary antibodies in blocking solution for 1h at room temperature (RT). Following washes (3 times for 10 min each with TBS-T), membranes were treated with ECL (Santa Cruz). Western blot visualization and densitometry analyses were performed using a Li-Cor Odyssey Fc imaging system and Image Studio. The following antibodies were used for western blot analysis in this study: β-actin (1:5000, #A5411, Sigma-Aldrich), tsg101 (1:1000, #ab83, Abcam), Vdac1 (1:1000, #ab154856, Abcam), mic60 (anti-misfiling) (1:1000, #10179-1-Proteintech), anti-Opa1 (1:1000, #27733-1-AP, Proteintech) HRP-conjugated goat-anti rabbit (1:5000, #111-035-144, Cedar lane), HRP-conjugated goat-anti mouse (1:5000, #115-035-003, Cedar lane). Unless stated otherwise, all immunoblots shown are representative of at least 3 biological replicates.

### Immunofluorescence

Cells cultured on glass coverslips were fixed overnight in 4% paraformaldehyde (Electron Microscopy Sciences) in 1X PBS at 4**°**C. Following 3 washes in 1X PBS cells were permeabilized with ice-cold methanol for 5min, washed and blocked in 5% BSA in PBS for 1h at RT. Cells were incubated with primary antibodies diluted in blocking solution for 1h at RT. Following 3 washes with PBS, secondary antibody incubations (1:1000) were performed for 1h at RT in blocking solution, followed by nuclear staining with DAPI (5 μg/ml). Coverslips were mounted using Dako Fluorescence Mounting Media (Agilent Technologies).

The following primary antibodies were used for immunofluorescence analysis: anti-TOM20 (Proteintech, #11802-1-AP, 1:500), anti-cytochrome C (Abcam, #ab110325, 1:500), anti-PDH (Abcam, #ab110333, 1:500), anti-Atp5L (Proteintech, #16483, 1:500), anti-Atp5α (Abcam, #ab14748, 1:500), anti-VDAC1 (Abcam, #ab154856, 1:500), anti-COXIV (Proteintech, #11242-1-AP, 1:500), anti-UCQCRC2 (Proteintech, #14742-1-AP, 1:500), anti-8OHdG (Santa Cruz, #393871, 1:100), anti-CD63 (Abcam, #ab8219, 1:100), anti-LBPA (Echelon Biosciences, #z-PLBPA, 1:500), anti-ALG2 (Proteintech, #12303-1-AP, 1:100). The following secondary antibodies were used for immunofluorescence analysis at 1:1000 dilution: Goat-anti-rabbit Alexa 647 (Invitrogen, # A21244), donkey-anti-rabbit Alexa 405 (Invitrogen, # A48258), goat-anti-rabbit Alexa 488 (Invitrogen, # A11011), goat-anti-mouse Alexa 488 (Invitrogen, # 11029), goat-anti-mouse Alexa 647 (Invitrogen, #A21235), goat-anti-rat Alexa 647 (Invitrogen, #A21247).

For Airyscan imaging, cells fixed with 4% paraformaldehyde (4**°**C, overnight) were quenched with 50mM ammonium chloride for 10min and washed with 1xPBS+5% FBS. Cells were permeabilized for 5min with ice cold methanol and blocked in 1xPBS+10% FBS for 1h at RT. Cells were incubated with indicated primary antibodies diluted in blocking solution for 1h at RT, washed with 1xPBS+5% FBS, followed by incubation with secondary antibodies (1:2000), washed and mounted onto slides.

### Microscopy

Unless stated otherwise, all fluorescent micrographs shown are high-resolution Airyscan images and time-lapse imaging was performed using Zeiss LSM880 Airyscan confocal using 63x/1.4 PlanApo objective. Spinning disc confocal images were acquired using 63x/1.4 NA objective using a Quorum spinning disc confocal microscope, consisting of an inverted fluorescence microscope (DMI6000B; Leica), an EM-CCD camera (Hamamatsu Photonics) and spinning disc confocal scan head. The equipment was controlled by Volocity acquisition software (Perkin Elmer). Z-stacks obtained were 300nm apart.

For live-cell imaging, cells were plated on glass coverslips and transfected with mito-BFP overnight. To label lysosomes, cells were incubated with dextran488 (25μg/ml) or dextran cascade blue (25μg/ml) overnight, washed and chased for an additional 3-5h in full media before imaging. 15min prior to live cell imaging cells were incubated with 100nM MitoTracker CMXRos in full media at 37**°**C. Media was removed and cells washed with dye-free media before moving to a pre-warmed microscope stage. High-resolution Airyscan images were acquired using a 63x Oil DIC M27 objective every 5 sec. Prior to analysis, raw image files were automatically processed into deconvoluted Airyscan images using the Zen software (Zeiss).

For analysis of lipid peroxidation, cells transfected with mito-BFP were incubated with MitoCLox followed by incubation with mitotracker DeepRed prior to imaging. Where indicated, cells were treated with 500μM H_2_O_2_ for 30min as positive control. Images were acquired using a Quorum spinning disc confocal microscope with excitation at 491 and 561 nm.

Confocal imaging for CLEM experiments was performed using a Leica SP8 confocal microscope where z-stack slices were set at 200nm.

#### Correlative light and electron microscopy

Plasmid DNA (LAMP1-GFP and mito-BFP) was electroporated into cells using the Neon system (Invitrogen). Cells were resuspended at 5×10^6^ cells in 100µl buffer R. 10 µl of cell/1 µg plasmid DNA mix was aspirated into a Neon pipette and electroporated in electroporation buffer ‘E’ at 1350 V for 30 ms with 1 pulse. Cells were then plated in glass bottom 35 mm MatTek dishes for confocal imaging and electron microscopy studies. For CLEM analysis of Tsg101 depleted cells, cells were treated with Tsg101 siRNA for 72h prior to electroporation of plasmid DNA. Before fixation, cells were incubated with 100nM MitoTracker Deep Red for 20min at 37^o^ C. Samples were then fixed by adding a mixture of 8% PFA in 200 mM HEPES buffer to culture medium (v/v) and incubated at RT for 15 min, then replaced with 4% PFA in 100mM HEPES for 30 min before imaging by confocal microscopy. Cells were imaged on a Leica SP8 confocal microscope using a 63X objective (oil, 1.4NA) and z-stacks were acquired 150nm/slice (1024x1024 resolution). A substack containing the event of interest was acquired at 2048x2048 resolution for subsequent CLEM analysis. Following fluorescence imaging, samples were transferred to 1% glutaraldehyde in 100 mM HEPES buffer.

#### Resin embedding

Fluorescently imaged samples were processed for CLEM in a Biowave Pro (Pelco, USA) with use of microwave energy and vacuum. Cells were twice washed in HEPES (Sigma-Aldrich H0887) at 250 W for 40 s, post-fixed using a mixture of 2% osmium tetroxide (Taab O011) 1.5% potassium ferricyanide (Taab, P018) (v/v) at equal ratio for 14 min at 100W power (with/without vacuum 20 ”Hg at 2-min intervals). Samples were washed with distilled water twice on the bench and twice again in the Biowave 250 W for 40 s. Samples were stained with 1 % aqueous uranyl acetate (Agar scientific AGR1260A) in distilled water (w/v) for 14 min at 100 W power (with/without vacuum 20 ”Hg at 2 min intervals) then washed using the same settings as before. Samples were dehydrated using a step-wise acetone series of 50, 75, 90 and 100 %, then washed 4x in absolute acetone at 250 W for 40 s per step. Samples were infiltrated with a dilution series of 25, 50, 75, 100 % Durcupan ACM® (Sigma-Aldrich 44610) (v/v) resin to propylene oxide. Each step was for 3 min at 250 W power (with/without vacuum 20 ”Hg at 30 s intervals). Samples were then cured for a minimum of 48 h at 60^o^C.

#### Sample trimming and transmission electron micrograph acquisition

Referring to grid coordinates, the sample block was trimmed, coarsely by a razor blade then finely trimmed using a 35^o^ ultrasonic, oscillating diamond knife (DiATOME, Switzerland) set at a cutting speed of 0.6 mm/s, a frequency set by automatic mode and a voltage of 6.0 V, on a ultramicrotome EM UC7 (Leica Microsystems, Germany) to remove all excess resin surrounding the ROI. Images were acquired using a 120 kv Tecnai G2 Spirit BioTwin (FEI company) with OneView Camera (Gatan).

#### CLEM image alignment

Fluorescent .LIF files were converted to tiff file format and liner adjustments made to brightness and contrast using FIJI (version 2.0.0-rc-69/1.52p). Images were aligned to EM micrographs with Icy 2.0.3.0 software (Institut Pasteur, France), using the ec-CLEM Version 1.0.1.5 plugin. No less than 10 independent fiducials were chosen per alignment for 2D image registration. When the fiducial registration error was greater than the predicted registration error, a non-rigid transformation (a nonlinear transformation based on spline interpolation, after an initial rigid transformation) was applied as previously described ^64^.

### Image analysis

#### Quantification of VDIMs

Unless stated otherwise, all quantifications were performed on maximum intensity projections of images acquired using a Spinning disc confocal microscope, using Image J (Fiji). OMM was labeled using anti-TOM20 antibodies and mitochondrial IMM labeled using mitotracker. ROIs were selected around cells and duplicated and a mask of each channel was generated. To do this, a manual threshold was applied to each channel and ‘erode’ command was applied to the mitotracker channel and ‘dilate’ command was applied to the TOM20 channel to expand the mask by 1 pixel. The TOM20 mask was subtracted from the mitotracker mask and the result was used to measure the number and size of VDIMs using “Analyze particles” plugin. Quantifications of recruitment of the different markers to VDIMs were performed manually using maximum intensity projections of Airyscan super-resolution images.

#### Time-lapse images

For fluorescence intensity measurements from time-lapse images, processed Airyscan images were analyzed using ImageJ (Fiji) software. ROIs were selected around entire mitochondria from where the VDIM pinched off at each time frame, and mitotracker intensity was measured. For analysis of VDIMs, dextran-labeled lysosomes were used to select the region of interest and the mean fluorescence intensity for indicated channels within the ROI was measured over time.

#### 3D-reconstruction

Surface reconstruction of fluorescence images acquired using Airyscan images as described above were processed using Imaris Bitplane 9.5 software using “surface” feature and default parameters (Oxford Instruments)

### Statistical analysis

Data shown are mean+/- SEM from independent biological replicates. As indicated, comparison between 2 groups was performed using unpaired 2-tailed Student’s *t*-test, and comparison between multiple groups was performed using one-way ANOVA with Tukey’s multiple comparison test using Prism software (GraphPad Software). *P* values calculated are indicated. 95% confidence interval was used to determine statistical significance and *P*<0.05 was considered to be statistically significant.

## Extended Data

## Supplementary Material

Supplementary Video 1

Supplementary Video 2

Supplementary Video 3

Supplementary Video 4

Supplementary Video 6

Supplementary Video 7

Supplementary Video 8

Supplementary Video 9

Supplementary Video 10

Supplementary Video 11

## Data Availability

All data supporting the conclusions of this study are available in the main text or extended figures. Source data for all the graphs are provided as separate excel files. Full versions of blots and gel are provided in Supplementary Figures.1-2.
